# Calibr improves spectral library search for spectrum-centric analysis of data independent acquisition proteomics

**DOI:** 10.1038/s41598-022-06026-9

**Published:** 2022-02-07

**Authors:** Jen-Hung Wang, Wai-Kok Choong, Ching-Tai Chen, Ting-Yi Sung

**Affiliations:** 1 Bioinformatics Program, Taiwan International Graduate Program, Academia Sinica, Taipei, 11529 Taiwan; 2 Institute of Information Science, Academia Sinica, Taipei, 11529 Taiwan; 3 Institute of Biomedical Informatics, National Yang Ming Chiao Tung University, Taipei, 11221 Taiwan; 4 Department of Bioinformatics and Medical Engineering, Asia University, Taichung, 41354 Taiwan; 5 Center for Precision Health Research, Asia University, Taichung, 41354 Taiwan

**Keywords:** Proteome informatics, Proteomic analysis

## Abstract

Identifying peptides and proteins from mass spectrometry (MS) data, spectral library searching has emerged as a complementary approach to the conventional database searching. However, for the spectrum-centric analysis of data-independent acquisition (DIA) data, spectral library searching has not been widely exploited because existing spectral library search tools are mainly designed and optimized for the analysis of data-dependent acquisition (DDA) data. We present Calibr, a spectral library search tool for spectrum-centric DIA data analysis. Calibr optimizes spectrum preprocessing for pseudo MS2 spectra, generating an 8.11% increase in spectrum–spectrum match (SSM) number and a 7.49% increase in peptide number over the traditional preprocessing approach. When searching against the DDA-based spectral library, Calibr improves SSM number by 17.6–26.65% and peptide number by 18.45–37.31% over two state-of-the-art tools on three different data sets. Searching against the public spectral library from MassIVE, Calibr improves state-of-the-art tools in SSM and peptide numbers by more than 31.49% and 25.24%, respectively, for two data sets. Our analyses indicate higher sensitivity of Calibr results from the use of various spectral similarity measures and statistical scores, coupled with machine learning-based statistical validation for FDR control. Calibr executable files including a graphical user-interface application are available at https://ms.iis.sinica.edu.tw/COmics/Software_CalibrWizard.html and https://sourceforge.net/projects/comics-calibr.

## Introduction

One of the major tasks in mass spectrometry (MS)-based proteomics is the identification of peptides and proteins^1^. Spectral library searching is a rising and promising research area and a complementary approach to conventional sequence database searching for identification^2,3^. As opposed to comparing each query MS2 spectrum against theoretical fragment ions of putative peptides, it compares the query spectrum against a library of experimental reference spectra for which the identifications are known, resulting in more sensitive and more rapid identification^4–8^. To date, spectral library searching has been successfully applied to a number of important research topics regarding phosphopeptides^9,10^, intact glycopeptides^11,12^, cross-linked peptides^13^, and missing proteins^14^.

Several spectral library search tools have been developed in the past years, for example, SpectraST^2,6,15^, Pepitome^16^, COSS^17^, and Epsilon-Q^18^. These tools rely on the same premise that the MS2 spectrum of a peptide under fixed conditions is a reproducible fingerprint of that peptide such that unidentified spectra under the same condition can be identified by spectral matching. They are particularly developed for DDA data analysis as the premise holds when the query spectra and spectral library are both based on data-dependent acquisition (DDA). In this paper, we study the spectral library search for data-independent acquisition (DIA) data, currently the increasingly adopted acquisition method for shotgun proteomics.

There are two types of schemes for analyzing DIA data. One is peptide-centric approach which searches the acquired data against assay libraries of MS2 spectra to retrieve groups of signals representing a specific peptide. The other is spectrum-centric approach which de-convolves the acquired DIA data into pseudo MS2 spectra for untargeted identification by conventional database searching same as for DDA data^19^. The peptide-centric approach shows higher sensitivity for peptide and protein identifications from DIA data^19,20^. In contrast, the spectrum-centric approach has potential of identifying new variant peptides and peptides with low intensities because pseudo MS2 spectra can cover peptides not included in the assay libraries^19,20^. Therefore, we work on spectrum-centric DIA data analysis and use spectral library searching, which shows high sensitivity in protein identification from DDA data, to perform identification from the pseudo MS2 spectra generated from DIA data. To validate the resulting spectrum–spectrum matches (SSMs), we consider using a machine learning-based validation tool. However, the pseudo MS2 spectra generated from DIA data can have significantly different spectral characteristics and varying signal qualities, leading to lower reproducibility of fragmentation patterns for peptides^21,22^. For such cases, existing spectral library search tools designed and optimized for comparing DDA spectra could suffer from decreased sensitivity. It is therefore necessary to develop a new spectral library search engine to address this issue.

In this study, we used DIA-Umpire 2.0^21^, a widely used spectrum-centric tool for DIA data analysis to generate pseudo MS2 spectra from DIA data sets. We used a public spectral library MassIVE^23^ and also a DDA-based spectral library constructed by SpectrsST using database search results of the same sample and spectra files in mzML format as input. Both spectral libraries are used to demonstrate the workflow and performance of spectral library searching for untargeted identification of DIA data. Furthermore, we use the DDA-based spectral library to optimize preprocessing of mass spectra and to utilize various scoring functions for better characterizing the similarities between the pseudo MS2 spectrum and a reference DDA spectrum for a given peptide, under the limitation that the two can have quite different patterns of fragment ions and signal qualities. To facilitate spectral matching based on our findings, we develop Calibr, a spectral library search tool which enhances the sensitivity of spectral library searching of pseudo MS2 spectra against a spectral library.

Similar to SpectraST, prior to spectrum–spectrum matching, Calibr performs a series of preprocessing steps on the query and library spectra, including modifying the intensity of each peak in a spectrum to the power of an exponent and down scaling of unannotated peaks’ intensities in a library spectrum. Our experiment results indicate the best parameters of these steps for pseudo MS2 spectra are different from those for DDA spectra. Calibr uses three types of measures to evaluate spectrum–spectrum comparison: spectral similarity, precursor property, and descriptive statistics. Spectral similarity consists of commonly used measures such as Xcorr^24^ and Kendall-Tau coefficient^16^, and a novel measure called library-centric cosine similarity. Precursor property includes the information such as mass difference and charge state of a precursor ion. Descriptive statistics includes the number of candidate spectra for which the comparison is made as well as measures such as mean and standard deviation of the dot product scores for these candidates. All these measures are compiled as a set of 19 features for each SSM, which are then taken as input for a machine learning-based validation tool—Percolator^25^—to generate the identification results under a false discovery rate (FDR) of 1% at both spectrum and peptide levels.

Both of our benchmark results of three DIA data sets using the public spectral library and the DDA-based spectral library show that Calibr yielded significant improvement over two compared popular tools in the identification coverage at both SSM and peptide levels. For example, in a HeLa cell data set and using the DDA-based spectral library for searching, Calibr achieved more than 23% and 18% improvements in validated SSMs and peptides, respectively, over the other two spectral library search engines. Notably, searching against the large public MassIVE spectral library, Calibr and the other two spectral library search tools achieved better identification than searching against the DDA-based spectral library. Moreover, our results also show that integration of database and spectral library search results of pseudo MS2 spectra further enhances spectrum-centric DIA data analysis. Calibr executable files and a graphical user-interface application, named CalibrWizard, which facilitates the spectral library search workflow in this study by Calibr and statistical validation, are available for download at https://ms.iis.sinica.edu.tw/COmics/Software_CalibrWizard.html and https://sourceforge.net/projects/comics-calibr.

## Methods and data sets

### Workflow of spectrum-centric identification of DIA data sets by searching pseudo MS2 spectra against a spectral library

**Figure 1:**
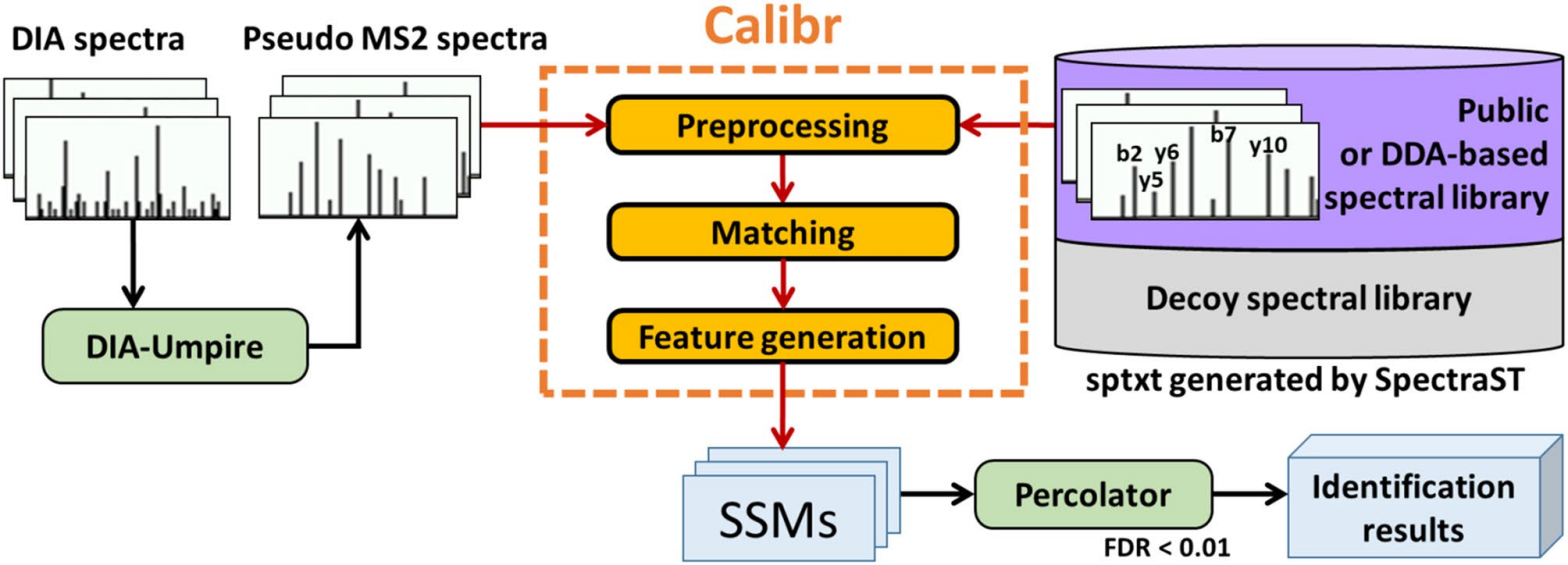
Spectrum-centric DIA data analysis workflow by spectral library searching. For DIA data, DIA-Umpire 2.0 is used for generating pseudo MS2 spectra. Calibr (or SpectraST or Pepitome) searches the pseudo MS2 spectra against a large public spectral library or a sample-specific DDA-based spectral library. The spectral library search results are then validated using Percolator, a machine learning-based validation tool.

The proposed workflow for DIA data analysis in this study adopts spectral library search approach on the pseudo MS2 spectra generated from DIA data sets by DIA-Umpire 2.0^21^ to search against a spectral library, as shown in Fig. 1. The spectral library used for the workflow can be constructed from DDA data sets of the same sample or obtained from public domain. SpectraST is used to build the consensus DDA spectral library and decoy spectral library from the validated search results of DDA data sets or from the external spectral library, Calibr is used to perform spectral library searches. Finally, Percolator^25^ is used to perform the validation of the spectral library search results using various measures provided by Calibr as features for machine learning.

### Generating pseudo MS2 spectra from DIA data sets

In spectrum-centric DIA data analysis, we used DIA-Umpire 2.0 to construct pseudo MS2 spectra from DIA spectra files, which are written to three groups of spectra files with decreased quality levels Q1, Q2, and Q3, respectively. All the spectra of three different quality levels were used as the input of spectral library search tools for spectral library searching.

### Constructing a consensus spectral library from the DDA data set

To construct a sample-specific spectral library from a DDA data set, we performed sequence database searches using three database search engines—Comet^24^, X!Tandem^26^, and MS-GF +^27^—against the UniProt human sequence database (uniport_human_20170615 FASTA file). The precursor m/z tolerance, number of missed cleavages, and post-translational modifications were set according to the publication of the DDA data set. Then we used PeptideProphet^28^ to validate the search results from each search engine passing FDR < 1% at the peptide-spectrum match (PSM) level. For each DDA data set, we then used SpectraST to construct a spectral library from the validated PSMs obtained from a search tool, yielding three spectral libraries from the three search tools. Subsequently, we used SpectraST to combine them and generated a consensus (target) spectral library (options –cJU -cAC) from the three spectral libraries of different database search tools. We also applied a quality filter for removing spectra at quality level 2 (default setting) (options -cAQ -cL2). Finally, we generated a decoy spectral library which was appended to the consensus library (options -cAD -cc -cy1). The concatenated target-decoy spectral library (still termed as spectral library for convenience) was used for subsequent spectral library searching. The sequence database searches, subsequent validation and library construction were performed using WinProphet^29^, a window-based software system compatible with the Trans-Proteomic Pipeline^30^, to construct and conduct the proteomics data analysis pipeline.

### Constructing a consensus spectral library from a large public spectral library

We downloaded a large public library (containing 2,154,269 spectra) in the .msp format from MassIVE^23^ (https://massive.ucsd.edu/ProteoSAFe/static/massive-kb-libraries.jsp). We used SpectraST to convert the msp file into splib and sptxt formats. Following similar procedure in constructing the spectral libraries from the DDA data sets, we constructed a consensus library, applied the quality filter and generated the decoy library appended to the consensus library. Then pseudo MS2 spectra can be searched against this large MassIVE spectral library.

### Calibr for spectral library searching

Calibr takes pseudo MS2 spectra files in mzML, mzXML, or MGF formats and a spectral library in sptxt format as input. It first preprocesses query spectra files and the spectral library, including adjustment of peak intensities and peak binning. Next, Calibr performs spectral library searching with a precursor tolerance of 0.5 Da, which is also adopted by the other compared library search tools. In particular, Calibr calculates various known spectral similarity measures. Among them, dot product is used to determine SSMs, while the remaining similarity measures and other metrics are used as features in Percolator for validation at the SSM and peptide levels.

### Preprocessing of spectra in query spectra files and spectral library

After taking query spectra files and a spectral library file as input, Calibr performs preprocessing on the spectra, similar to SpectraST. First, we implemented intensity power (IP), in which the intensity of each peak in a spectrum is modified to the power of an exponent. IP with an exponent smaller than 1 can reduce the contribution of dominating high peaks in similarity scoring of spectrum matching. Second, we implemented the unassigned-peak scaling (UPS), in which the intensities of all the peaks without ion-type annotation in a library spectrum are multiplied by a factor less than 1. UPS can relatively increase the contribution of annotated peaks for spectrum matching. Third, we implemented the signal-to-noise filter (SNfilter), which is called dynamic range in SpectraST. Given a factor, say 1000 for example, of SNfilter, Calibr determines a peak intensity threshold for each query spectrum as the intensity of the highest peak divided by the factor. All the peaks with intensities larger than the determined threshold are kept in the spectrum.

After processing the peaks in a spectrum with the IP, UPS and SNfilter operations, Calibr performs binning to reduce spectrum complexities. All the peaks are assigned to 1 Th-sized bins by rounding the peak m/z value to an integer. Calibr supports two strategies to determine the intensity of a bin, one by the intensity of the highest peak in the bin (denoted as Max) and the other by the sum of peak intensities in the bin (denoted as Sum). Henceforth, we use the term “intensity” to represent the intensity of a bin and the term “peak” to mean the peak of a bin for convenience.

After binning, Calibr provides Spillover as a user’s option on only query spectra, on only library spectra, or on both, slightly different from SpectraST which performs on both query and library spectra when Spillover is enabled. It aims to improve spectrum matching by including two peaks in a query spectrum and a library spectrum, respectively, having very close m/z values but assigned to two neighboring bins for similarity calculation. When the binning strategy is Max, for each bin Spillover assigns half of its intensity to replace the intensity of its neighboring bin at left or right which has a smaller intensity than the intensity to be assigned. When the binning strategy is Sum, for each bin Spillover adds half of its original intensity to both of its neighboring bins.

Then Calibr performs normalization on all the intensities in a spectrum so that the squares of normalized intensities sum up to 1. Normalized intensities are used for subsequent calculation of various similarity measures between a query spectrum and a library spectrum; particularly, the dot product of normalized intensities from two spectra is also their cosine similarity.

### Spectrum–spectrum similarity measures for Calibr spectral library searching

Calibr calculates various similarity measures between a query and a library spectrum, including dot product used in SpectraST, Pearson correlation coefficient (PCC), Comet’s cross correlation (Xcorr), Kendall-Tau coefficient (KT) and hypergeometric test score (HGT) used in Pepitome, as well as dot product-related scores used in SpectraST, such as deltaD, dotBias, and Fval (see Table 1). In addition, we particularly designed a new score, called *library-centric cosine similarity* and denoted by libc_cosSim, where only the positive-intensity bins of the query spectrum matched to the library spectrum are used to calculate cosine similarity. The formula of all the similarity scoring measures are listed in Supplementary Table S1. When searching the pseudo MS2 spectra file(s) of a DIA data set against the constructed spectral library, for each query spectrum Calibr reports the SSM with the highest dot product.

**Table 1:** Features provided by different spectral library search engines for SSM and peptide validation using Percolator.

	Spectral library search engine		
	Calibr	SpectraST	Pepitome
Precursor and spectrum property	Mass difference	Mass difference	Mass difference
	Precursor mz difference	Precursor mz difference	
	Charge state	Charge state	Charge state
	Spectrum quality level	Spectrum quality level	Spectrum quality level
Similarity measure	Dot product	Dot product	Hypergeometric test score
	deltaD	deltaD	Kendall-Tau coefficient
	dotBias	dotBias	Mvh
	Fval	Fval	mzFidelity
	Penalty (function of dotBias)		
	Second best dot product		
	Xcorr		
	Library-centric cosine similarity		
	Kendall-Tau coefficient		
	Hypergeometric test score		
	Pearson correlation coefficient		
Statistics	Number of hits	Number of hits	num_matched_ions
	Mean of dot products of the hits	Mean of dot products of the hits	tot_num_ions
	SD of dot products of the hits	SD of dot products of the hits	kendallPVal
	*p* value of the best hit	*p* value of the best hit	
		first_non_homolog	
		lib_probability	
		lib_num_replicates	

### Validation of SSMs by Percolator

All the SSMs reported from spectral library searching require further validation to discriminate positive SSMs from negative SSMs passing a specific FDR. When we use only a single tool for searching, we consider using Percolator for validation because it allows various features to be used for machine learning, instead of conventionally using the single feature which determines the SSMs for validation. For Calibr, in addition to the above-mentioned spectral similarity measures, metrics related to “precursor and spectrum property” and “statistics” are also considered as features for Percolator (Table 1). Features related to precursor ion and spectrum property are as follows: mass difference, precursor m/z difference, precursor charge state, and spectrum quality level. The last two features are categorical features, which are encoded by one-hot encoding with a vector of length five for charge state of 1 to 5 and a vector of length three indicating the spectrum quality level of Q1, Q2, or Q3 reported by DIA-Umpire 2.0, respectively. The statistics-related features include descriptive statistics for the distribution of dot products between the query spectrum and all the candidate library spectra with precursor m/z within the tolerance, e.g., number of these candidate library spectra (hits_num), mean of the dot products of the hits (hits_mean), standard deviation of the dot products of the hits (hits_stdev), and *p* value of the top hit (Pval) assuming that dot products have a normal distribution. After Percolator validation with all these features, SSMs and peptides with FDR < 1% are reported as validated results.

To validate the search results of DIA data sets from the other two spectral library search tools—SpectraST and Pepitome, we also extracted all metrics of spectrum–spectrum comparison from their SSMs as features for Percolator similar to Calibr’s features. The features used in Percolator validation provided by Calibr, SpectraST, and Pepitome, respectively, are summarized in Table 1, where Calibr has the largest number of features (19), followed by SpectraST (15) and Pepitome (10).

### Enhancement of identification by combining spectral library search and sequence database search results

To enhance identification coverage in spectrum-centric DIA analysis, we considered integrating the results of spectral library searching and sequence database searching on pseudo MS2 spectra files. Calibr (or SpectraST) was used to perform spectral library searching against the MassIVE spectral library as described above. We conducted a simple workflow based on Trans-Proteomic Pipeline^30^ to first perform database searches on the pseudo MS2 spectra against the UniProt database (uniport_human_20170615 FASTA file) using Comet, MS-GF + and X!Tandem and then combine their search results with Calibr’s results. When performing database searches, the search parameters of the three database search engines were set the same as those for their paired DDA data sets specified in their original publications of the data sets^21,31^. Since it is still difficult to develop common features across different search engines for machine learning based validation, we used PeptideProphet to validate the search results satisfying FDR < 1% from each database and spectral library search engine. The four validated results from four search engines—Comet, X!Tandem, MS-GF +, and Calibr (or SpedtraST)—were then combined and refined using iProphet^32^ for the final identification results.

### Coupled DDA and DIA data sets

We downloaded three pairs of MS spectra data sets acquired from both DDA and DIA modes on the same samples, respectively. All the downloaded .raw files were converted to mzXML files using MSConvert^33^.

The first pair of MS data sets were acquired from analyzing Hela cells and were downloaded from ProteomeXchange Consortium^34^ with identifier PXD003179^21^. We used the three DDA raw files and the three DIA raw files acquired with an isolation window of 10 Da. The DDA and DIA data sets are termed helaDDA and helaDIA, respectively.

The second pair of MS data sets were acquired from analyzing HEK293 cells on Orbitrap Lumos and were downloaded from ProteomeXchange Consortium with identifier PXD009246^31^. We used the three DDA raw files and the three DIA raw files, each of which were generated with 2000 ng sample amount. These two data sets are termed samonDDA and samonDIA, respectively.

The third pair of data sets were acquired from phosphoproteomic analysis of MCF7 cell line on a Q-Exactive mass spectrometer and were downloaded from ProteomeXchange Consortium with identifier PXD003344^35^. The DDA raw files and DIA raw files in technical quadruplicate are termed MCF7phosDDA and MCF7phosDIA, respectively.

The file names and the URL of the above data sets are listed in Supplementary Table S2.

## Results and discussion

### Pseudo MS2 spectra generation of DIA data and spectral library construction of DDA data

By using DIA-Umpire 2.0, the helaDIA, samonDIA and MCFphosDIA data sets yielded 553839, 889368 and 268061 pseudo MS2 spectra, respectively, which include spectra of all the three quality levels (Q1, Q2, and Q3) and can be searched by sequence database search tools and spectral library search tools. The numbers of spectra of Q1, Q2, and Q3 for each data set are listed in Supplementary Table S3.

We first used DDA-based spectral libraries to perform spectral library searching on pseudo MS2 spectra generated from DIA data sets. SpectraST was used to construct spectral libraries from the sequence database search results of the paired DDA data sets. The spectral libraries generated from helaDDA, samonDDA and MCF7phosDDA contained 34699, 40603 and 10548 target spectra, corresponding to 30799, 33559, and 7128 distinct stripped peptides (i.e., regardless of charge state and modification), respectively, where each target spectrum yielded a decoy spectrum in the spectral library.

### Performance comparison of different spectral library search tools to search against the DDA-based spectral libraries

We compared the performance of Calibr and two popular tools—SpectraST and Pepitome—on the pseudo MS2 spectra files of helaDIA, samonDIA, and MCF7phosDIA by searching against the corresponding DDA-based spectral libraries. All the three tools adopted a precursor m/z tolerance of 0.5 Da on the pseudo MS2 spectra files of the three DIA data sets. Calibr adopted an IP of 0.33, a UPS of 0.4, and binning with the “Max” strategy to preprocess the MS2 spectra files and then performed searching. SpectraST adopted an IP of 0.5 (default setting) and a UPS of 0.2 suggested in the original paper of SpectraST^6^ for preprocessing of the MS2 spectra files and adopted default parameter setting for searches. For Pepitome, we used its entire default settings for preprocessing and searching. All the SSMs obtained from each tool were then validated by Percolator, where the features used for each search tool are listed in Table 1.

**Figure 2:**
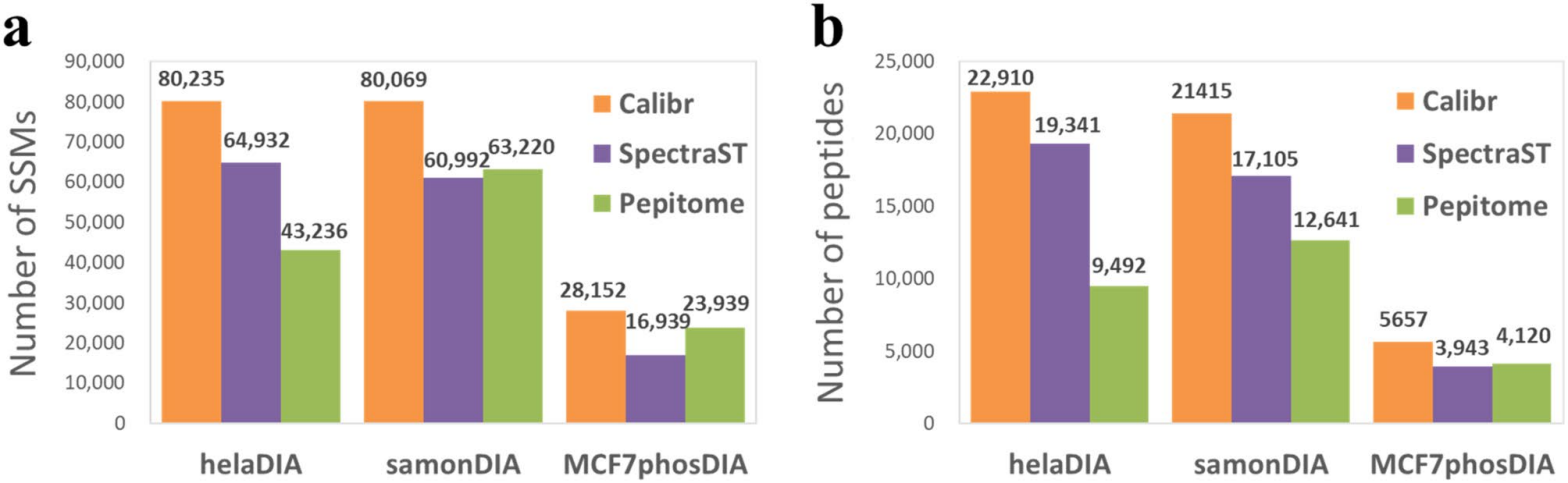
Identification results of three spectral library search engines on the helaDIA, samonDIA, and MCF7phosDIA data sets using the respective DDA-based spectral libraries. (**a**) The number of validated SSMs. (**b**) The number of validated peptides.

From the three DIA data sets, the identification numbers passing FDR < 1% obtained by Calibr, SpectraST, and Pepitome are shown in Fig. 2 and Supplementary Table S4. Notably, Calibr achieved the highest number of validated SSMs and peptides in all the MS2 spectra files of all the three DIA data sets. In terms of SSM number, Calibr improved at least 23.56%, 26.65%, and 17.60% on helaDIA, samonDIA, and MCF7phosDIA, respectively (Fig. 2a). In terms of peptide number, Calibr improved at least 18.45%, 25.20%, and 37.31% on helaDIA, samonDIA, and MCF7phosDIA, respectively (Fig. 2b).

As a benchmarking of computing resources, we recorded the processing time and the maximum memory usage used by the three tools for searching helaDIA data set and calculating various features for the SSMs. Calibr, written in C#, took 8 min 23 s and 4516 MB. SpectraST and Pepitome, both written in C++, took 5 min 20 s with 751 MB, and 8 min 25 s with 1910 MB, respectively. The results showed that SpectraST was most efficient among the three tools. Calculating additional similarity features such as Xcorr and library-centric cosine similarity resulted in Calibr’s larger maximum memory usage and longer processing time.

### Parameter optimization for spectrum preprocessing in Calibr

**Figure 3:**
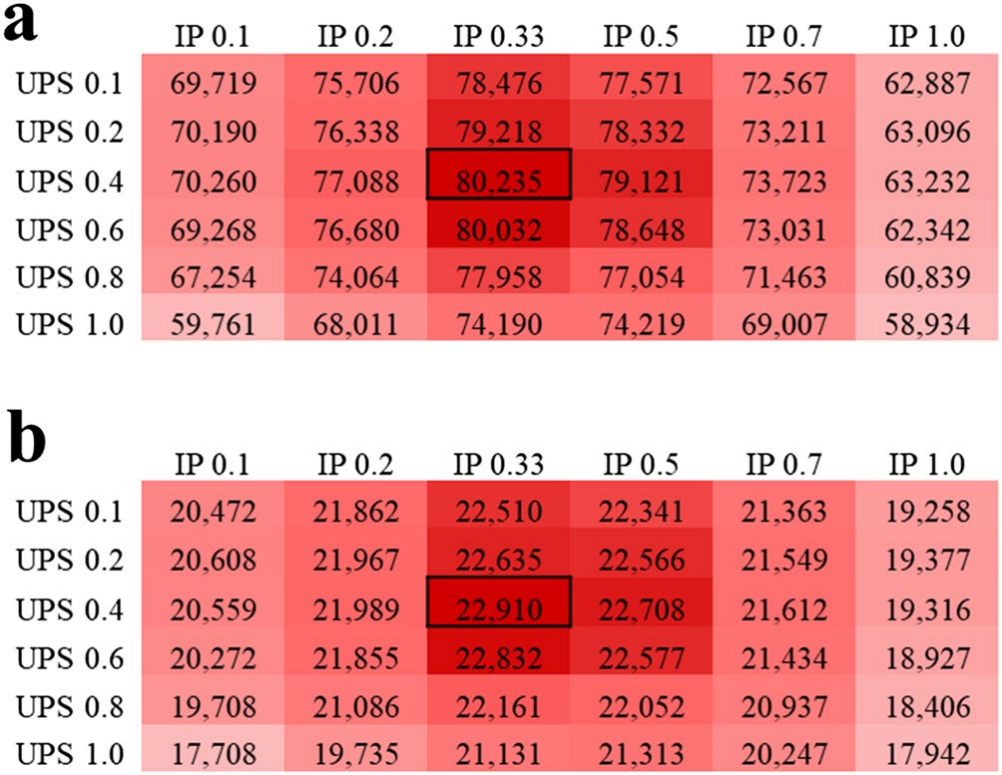
Comparison of different combinations of intensity power (IP) and unassigned peak scaling (UPS). The comparison is based on identification results of searching the helaDIA data set against the DDA-based spectral library using Calibr. (**a**) The number of validated SSMs. (**b**) The number of validated peptides. Validation was performed using Percolator. The largest value is marked by a black box.

In the preprocessing steps, IP and UPS are two influential factors that can affect the search results. In order to optimize the parameter setting for IP and UPS, we considered six values between 0 and 1—0.1, 0.2, 0.33, 0.5, 0.7 and 1 for IP and 0.1, 0.2, 0.4, 0.6, 0.8 and 1 for UPS. It resulted in 36 combinations of IP and UPS values for preprocessing a query data set and its corresponding spectral library. We used the pseudo MS2 spectra files to search against the respective DDA-based spectral libraries for parameter optimization, where the IP and UPS values achieving the best identification results were selected as default setting. Figure 3 shows the numbers of validated SSMs and peptides from pseudo MS2 spectra of helaDIA data set for all combinations of IP and UPS; the samonDIA and MCF7phosDIA data sets also showed similar optimized values (with slight difference) of IP and UPS as the helaDIA data set (see Supplementary Figs. S1 and S2). Hence, an IP of 0.33 and a UPS of 0.4 were selected for Calibr in this study. Using such settings, Calibr generated 80235 SSMs and 22910 peptides for helaDIA data set (the two entries in black boxes of Fig. 3). It is worth noting using the default settings of SpectraST, i.e., an IP of 0.5 and a UPS of 1.0, resulted in 74219 SSMs and 21313 peptides. Compared to such settings, the optimized IP and UPS generated an 8.11% increase in SSM number and a 7.49% increase in peptide number. The results indicate that pseudo MS2 spectra have different spectral characteristics to typical DDA spectra, and the optimized IP and UPS can significantly improve the identification coverage of SSMs and peptides for pseudo MS2 spectra.

### Investigating factors affecting the results of spectrum–spectrum matches among different spectral library search tools

**Figure 4:**
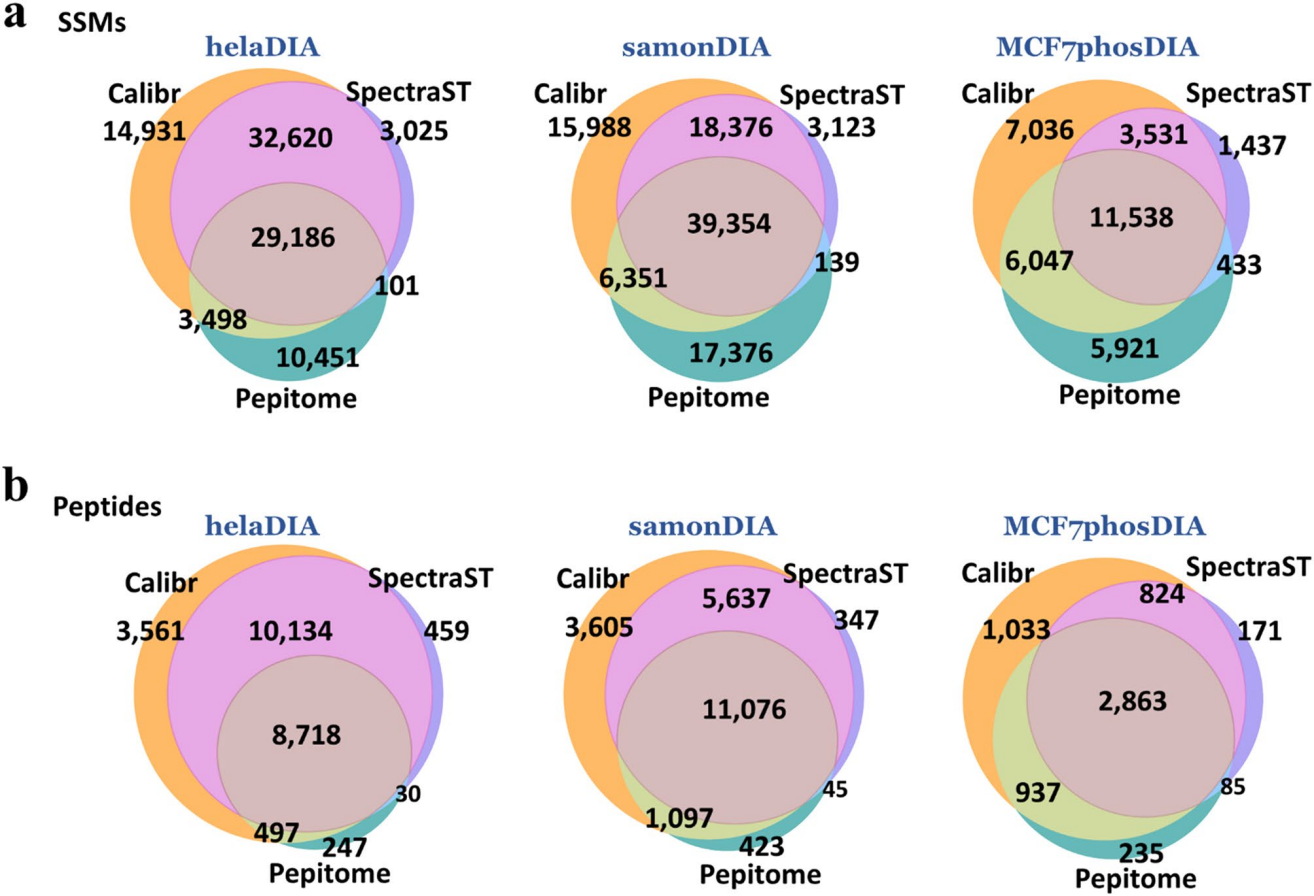
Comparison of identification results of different spectral library search engines searching against the respective DDA-based spectral libraries on the helaDIA, samonDIA, and MCF7phosDIA data sets. (**a**) Venn diagram of validated SSMs. (**b**) Venn diagram of validated peptides.

To investigate factors possibly affecting validated SSMs, we first compared the overlapping of validated identifications among the three spectral library search tools searching against the DDA-based spectral library as shown in Fig. 4. The Jaccard index^36^, defined as the size of the intersection divided by the size of the union of the two circles, was calculated at both SSM and peptide levels to represent the similarity between two methods, as illustrated in Supplementary Table S5. The Jaccard indices between Calibr and SpectraST are mostly larger than or equal to those between Calibr and Pepitome for both SSM and peptide levels in all three data sets. The results suggest similar preprocessing algorithms of Calibr and SpectraST and their common features (such as dot product, Fval, and deltaD) for SVM learning in Percolator lead to more common SSMs and peptides. Pepitome has quite different features and preprocessing algorithms to SpectraST, and it therefore shared relatively less common SSMs to both SpectraST and Calibr.

We used the SSMs commonly identified by the three search tools to examine the similarity of Percolator’s SVM scores between any two tools. The pairwise similarity of SVM scores among the three search tools are shown as correlation plots in Supplementary Fig. S3. For the helaDIA, samonDIA, and MCF7phosDIA data sets, the highest correlations of SVM scores were obtained between Calibr and SpectraST, i.e., 0.82, 0.82, and 0.68, respectively. The lowest correlations for the three DIA data sets were always between SpectraST and Pepitome, i.e., 0.56, 0.55, and 0.41 for the three DIA data sets, respectively. In short, Calibr and SpectraST showed higher correlations, and Pepitome showed significantly lower correlations to any of the other two.

**Figure 5:**
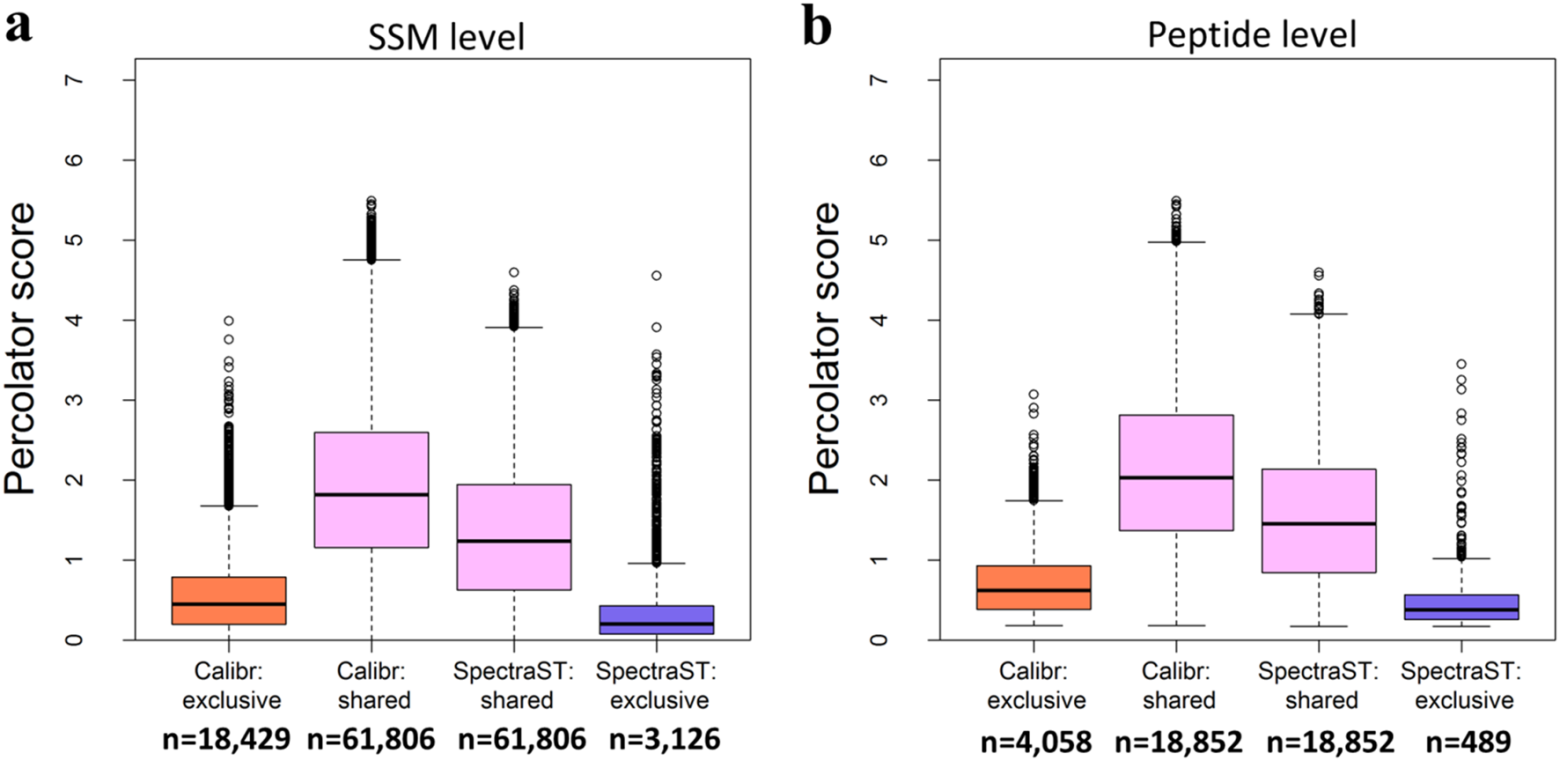
Distribution of Percolator SVM scores obtained by Calibr and SpectraST when searching the helaDIA data set against the DDA-based spectral library. (**a**) Scores of validated SSMs. (**b**) Scores of validated peptides. The SSMs and peptides are grouped into commonly obtained by SpectraST and Calibr (pink boxes) and exclusively obtained by one search engine (orange box for Calibr and purple box for SpectraST). The numbers indicate the numbers of SSMs and peptides.

Since Calibr and SpectraST had more similar Percolator’s SVM scores on the three DIA data sets, we then examined the scores of SSMs obtained by Calibr and SpectraST. For the helaDIA data set, the SVM scores of SSMs and peptides commonly identified by both tools and exclusively identified by either one are shown as box plots in Fig. 5. The exclusive SSMs obtained by either search engine generally have lower scores than the SSMs commonly obtained by both tools, i.e., the pink box is higher than the orange box for Calibr, and the pink box is higher than the blue box for SpectraST (Fig. 5a). Percolator’s SVM scores of peptides also show similar trends (Fig. 5b). Furthermore, examining the common SSMs identified by Calibr and SpectraST, the distribution of the SVM scores of Calibr was higher than that of SpectraST. To explore possible reasons, by replacing individual optimized parameters for spectra preprocessing or omitting exclusive features generated by Calibr for validation, we observed that optimized parameters for preprocessing spectra in Calibr play a more important role in spectral library search, resulting in higher SVM scores, than generated features for validation (Supplementary Fig. S4). The distribution difference can also be observed when searching samonDIA and MCF7phosDIA data sets, as shown in Supplementary Figs. S5 and S6.

### Discovering influential features for discriminating positive and negative SSMs

Calibr generates various features for each SSM, which are used by a machine learning approach such as Percolator to discriminate positive and negative SSMs for FDR control. It is important to discover crucial features for machine learning. For this purpose, we iteratively applied a leave-one-out strategy to examine each feature’s contribution by excluding it from all the features for Percolator. Let SSM_all and SSM(*f*) denote the numbers of validated SSMs passing Percolator validation using all the features and using all the features except the feature *f*, respectively. Then we define the contribution of the feature *f*, cont_SSM(*f*), as [SSM_all-SSM(*f*)] / SSM_all. Similarly, we can define the contribution of a feature *f* in terms of identified peptides, cont_Pep(*f*), where Pep_all and Pep(*f*) are defined similar to SSM_all and SSM(*f*). Here, we used SSMs obtained from searching against the DDA-based spectral library for the analysis.

**Figure 6:**
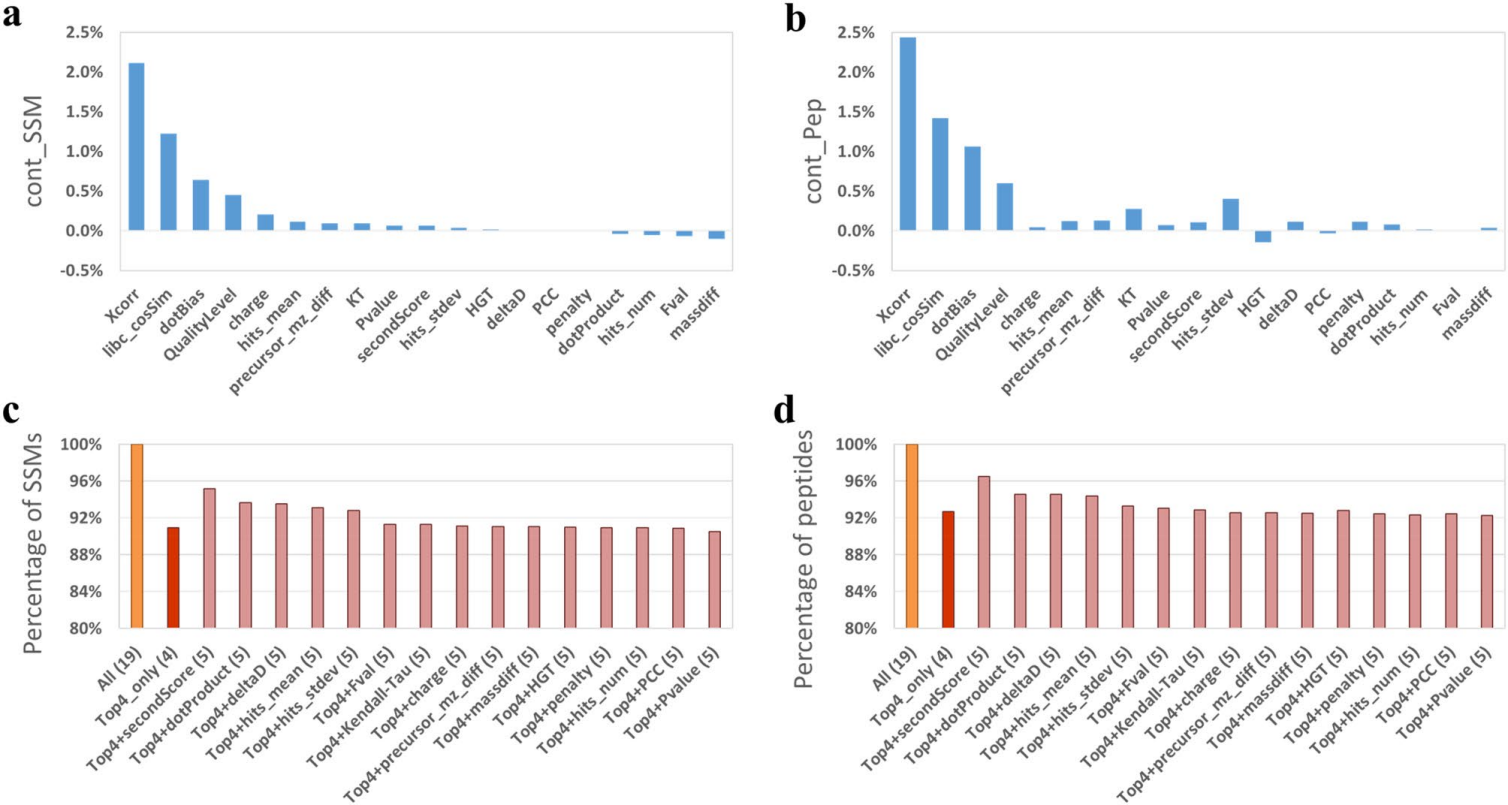
Evaluations of each single feature and different feature combinations used in Percolator validation on the helaDIA data set. Each single feature is evaluated by its contribution: (**a**) cont_SSM, in terms of validated SSMS, (**b**) cont_Pep in terms of validated peptides, which are defined as the percentage of decreased SSMs and peptides, respectively, of Calibr’s search results by excluding exactly the single one feature (leave-one-out) in validation. Different “top four-plus-one” features are evaluated with respect to using all the features (as 100%): (**c**) evaluation at the SSM level, (**d**) evaluation at the peptide level. “Top four-plus-one” features include the top four features—Xcorr, libc_cosSim, dotBias and QualityLevel—and one of the remaining features.

As mentioned earlier, Calibr generates 19 features, including various scores and two categorical features, i.e., charge state of 1–5 and spectrum quality level Q1, Q2 or Q3 from DIA-Umpire 2.0. Both categorical features are encoded by one-hot encoding with binary vectors of dimension five and three, respectively. The leave-one-out results on the helaDIA data set for feature evaluation are shown in Fig. 6a, b and Supplementary Table S6, in which our proposed novel libc_cosSim is the second important feature, and Xcorr, dotBias, and QualityLevel are the other top four important features in evaluations using validated SSMs and validated peptides in terms of cont_SSM and cont_Pep, respectively. In addition, the samonDIA data set also has the same top four features as the helaDIA data set.

The top two features, Xcorr and libc_cosSim, both employ the formula similar to dot product, where Xcorr calculates dot product on a pair of mass spectra with peaks shifted multiple times, and libc_cosSim proposed in this study calculates cosine similarity with a reduced norm for query spectra. Both Xcorr and libc_cosSim were found very useful for validation. Furthermore, they can be considered for being used as the scoring function in spectrum–spectrum matching. Particularly for our proposed libc_cosSim, Supplementary Fig. S7 shows pseudo MS2 spectra in the helaDIA data set that yielded the SSMs having relatively high libc_cosSim but having relatively low dot product, Xcorr, HGT, KT, and PCC and still passing FDR of 1%. The scoring function dotBias proposed in SpectraST is the third important feature in the leave-one-out evaluation, whereas SpectraST transforms dotBias into a penalty term in the formula of Fval score to be used by PeptideProphet for validation. Though Calibr also generates the penalty score and the Fval score as features for Percolator validation, they do not show great contribution in this evaluation. The QualityLevel feature, which indicates the quality level of a pseudo MS2 spectrum, also contributes important information for validation.

It is noted that dot product is a less important feature; excluding dot product from Percolator validation does not significantly affect the numbers of identification. This phenomenon can be explained by the use of dot product in the searching step that yields the initial order of SSMs. Therefore, the initial distributions of positive SSMs and negative SSMs are highly associated with the dot product feature. In other words, the composition of positive and negative SSMs has contained the information provided by dot product. In order to further separate the positive and the negative SSMs, we need to consider other features that have complementary information to dot product.

Since Xcorr, libc_cosSim, dotBias, and QualityLevel are shown to be the top four features for validation, we further examined the effect of the remaining features by conducting an iterative “top four-plus-one” evaluation, i.e., using the top four features and one of the remaining feature in turn for validating Calibr’s search results of helaDIA data set. The results are shown in Fig. 6c, d and Supplementary Table S7, where the percentages of validated SSMs and validated peptides using top four features only (red bar) and top four-plus-one features with respect to using all the features (as 100%) are shown for comparison. Using only the top four features in validation achieved 90.92% and 92.67% of validated SSMs and peptides obtained from using all features, respectively. Using one more feature in addition to the top four features can increase validated SSMs and peptides over using only the top four features. Particularly, with additional the second-best dotProduct score (secondScore) improved the identification by 4.27% for SSMs and 3.82% for peptides. Adding dotProduct, deltaD, hits_mean, and hits_stdev, respectively, also improved the SSMs by 1.87–2.73% over using only the top four features for validation. The above five features very likely harbor complementary information to the top four features in discriminating the positive and negative SSM distributions. However, when adding one of the remaining ten features, no obvious improvement was observed compared to using only the top four features, and even some of them showed decreased performance. It revealed that each of these ten features was less informative to the validation described by the top four features. For different data sets, it is very likely that the order of feature contribution becomes different from the order for helaDIA in the leave-one-out and four-plus-one evaluations. But it is noted that using all features, i.e., including measures which have seemingly less importance in feature ranking, can in fact benefit the SVM training procedure of Percolator and improve the coverage of identification. This is also one of the major reasons why Calibr outperformed the compared tools.

### Performance comparison of different tools searching pseudo MS2 spectra against a large-scale public spectral library

**Figure 7:**
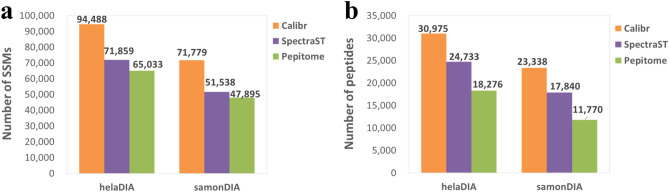
The identifications by three search engines searching pseudo MS2 spectra against MassIVE library. (**a**) The number of validated SSMs. (**b**) The number of validated peptides.

As described in the “Methods and data sets” section, we processed the MassIVE library, which originally contained 2154269 spectra from 1100505 peptides. After preprocessing and applying a quality filter, 2113381 consensus spectra from 1094958 peptides remained and decoy spectra were generated accordingly; both types of spectra were concatenated to form the MassIVE spectral library for search. We then used the three spectral library search tools to search the pseudo MS2 spectra of helaDIA data set and samonDIA data set against the MassIVE spectral library, and search results were further validated by Percolator such that all the peptides and SSMs have an FDR no greater than 1%. MCF7phosDIA data set was not analyzed in this section because the MassIVE library is not particularly designed for phosphopeptides. When compared with existing tools, Calibr improved SSM numbers by at least 31.49% and 39.27% on helaDIA and samonDIA, respectively, as illustrated in Fig. 7. Calibr improved peptide numbers by at least 25.24% and 30.82% on helaDIA and samonDIA, respectively.

We further compared the results of searching against the MassIVE spectral library (Fig. 7) with those from searching against DDA-based library (Fig. 2) to investigate the effect of the significant augmentation of a spectral library (an approximately 60-fold increase in spectrum number, an approximately 35-fold increase in peptide number), and the results are shown in Supplementary Table S8. For the helaDIA data set, searching against the MassIVE spectral library yielded improvements of 17.76%, 10.66%, and 50.41% in SSM numbers for Calibr, SpectraST, and Pepitome, respectively. Searching against the MassIVE spectral library yielded improvements of 35.20%, 27.87%, and 92.54% in peptide numbers for Calibr, SpectraST, and Pepitome, respectively. As expected, searching against a considerably larger spectral library led to much higher identification coverage. The relatively larger improvement of Pepitome revealed it benefited the most compared to other methods. Nevertheless, Calibr still generated the largest number of SSMs and peptides. For the samonDIA data set, Calibr and SpectraST showed improvements of 8.98% and 4.30% in the number of peptides, respectively, despite the fact that both of them have decreased SSM numbers.

These results suggest that the spectrum-centric analysis of DIA data can be greatly improved by searching against a significantly larger public spectral library as an alternative to searching against the sample-specific DDA spectral library, except for the data sets from samples with specific enriched modified peptides such as phosphopeptide enrichment. With ever-growing public spectral libraries available such as MassIVE, the spectral library searching approach can play an important role in spectrum-centric DIA data analysis.

### Enhancing DIA identification by integrating spectral library searching and sequence database searching

**Figure 8:**
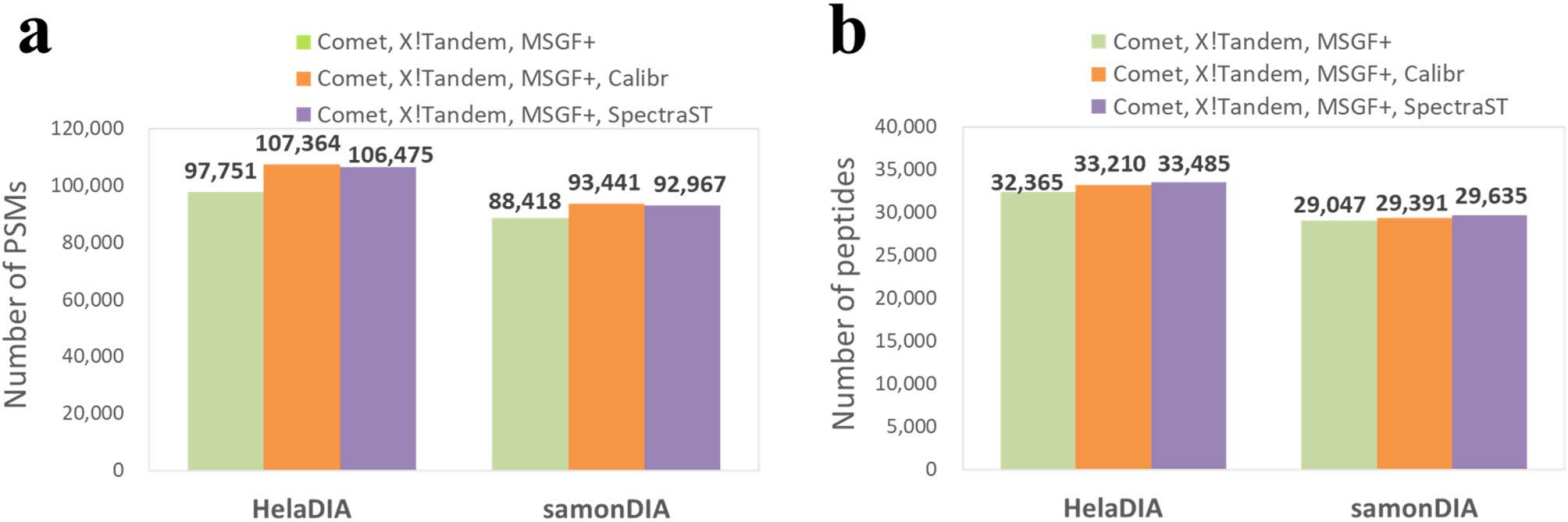
Identifications with FDR < 1% in combined results of three database search engines and one spectral library search engine. The search results were validated using PeptideProphet and combined using iProphet. (**a**) The numbers of validated PSMs. (**b**) The numbers of stripped peptides.

Currently, spectrum-centric DIA data analysis uses pseudo MS2 spectra generated by DIA-Umpire to perform sequence database searching, which is widely used for peptide identification. To further enhance spectrum-centric DIA data analysis, we combined the results of spectral library searching (against a public spectral library) and sequence database searching (see Fig. 8). As described in “Methods and data sets” section, we used PeptideProphet (instead of Percolator) to validate the search results from each database and spectral library search engine and then use iProphet to combine multiple PeptideProphet results.

For helaDIA data set, combined database search results of Comet, X!Tandem and MS-GF + yielded 97751 PSMs (32365 peptides), and using Calibr to search against the MassIVE spectral library yielded 67889 PSMs (21680 peptides) (Supplementary Fig. S8a). Integrating database search results and Calibr’s search results yielded 107364 PSMs (33210 peptides), an improvement of 9.83% and 2.61% in PSMs and peptides, respectively, over the database search results from the three search engines. Besides, using SpectraST to search against the MassIVE spectral library yielded 67293 PSMs (22327 peptides). Integrating database search results and SpectraST’s search results yielded 106475 PSMs (33485 peptides), an improvement of 8.92% and 3.46% in PSMs and peptides, respectively, over the database search results (see Supplementary Fig. S8b).

For samonDIA data set, the three database search engines yielded 88418 PSMs (29047 peptides). Calibr yielded 49201 PSMs (16625 peptides) and SpectraST yielded 47082 PSMs (16565 peptides), as shown in Supplementary Fig. S8c and d. Combining the database search results and Calibr’s results yielded 93441 PSMs (29391 peptides), which improves 5.68% (1.18%) from using database search engines. Combining the search results of database search engines and SpectraST yielded 92967 PSMs (29635 peptides), an improvement of 5.14% (2.02%) over database search results.

The above results show that spectrum-centric DIA data analysis can be enhanced by integrating spectral library search results with the database search results. We also noted that integrating Calibr’s search results with the database search results were just comparable to integrating SpectraST’s results with the database search results in this TPP-based workflow without using Percolator for validation. When a machine-learning based validation tool is available for integrating or developing multiple unified measures across different search engines as features for validation, the spectrum-centric DIA data analysis can be further improved.

### Graphical user interfaces for Calibr spectral library searching

We used C# language to implement Calibr as a command-line software tool on the Windows system. To convert Calibr’s search results to the format for validation by Percolator, we also implemented a conversion tool, called PercolatorTsvConverter. In addition, we provide a graphical user interface application, named CalibrWizard, which integrates Calibr and PercolatorTsvConverter, and can use the function of Percolator for validation (see Supplementary Fig. S9). Users can run the C# programs on the Windows system, and also on Linux or other platforms using Mono^37^. With the wizard, users can conveniently set the parameters of Calibr, perform spectral library searching, convert the search result files in pep.xml format into tab-delimited format (as input to Percolator), and use Percolator to perform validation. The validated search results are outputted as one .xml file of complete identification results and multiple files in tab-delimited format of protein, peptide and SSM tables.

## Conclusion

We have developed a command-line spectral library search tool, called Calibr, and a Windows-based friendly user interface application, called CalibrWizard, to seamlessly perform spectral library searches on pseudo MS2 spectra generated using DIA-Umpire, followed by Percolator validation for spectrum-centric analysis of DIA data. In this study, we also showed that optimized IP and UPS for spectrum preprocessing achieve 8.11% and 7.49% improvements in SSM and peptide numbers compared to the default values of SpectraST. Calibr provides various spectral similarity measures, including the novel library-centric cosine similarity (libc_cosSim). Our leave-one-out feature analyses showed Xcorr, libc_cosSim, dotBias, and QualityLevel are the top four dominating features. However, incorporating other seemingly less important features can still considerably improve the number of SSMs and peptides, because they benefit the machine-learning based validation tool, Percolator. When searching against the DDA-based spectral library, Calibr increased SSM number by 17.6–26.65% and peptide number by 18.45–37.31% compared to two state-of-the-art tools on three different data sets. When searching against a considerably larger public spectral library from MassIVE, Calibr improved the numbers of SSMs and peptides by more than 31.49% and 25.24%, respectively, compared to state-of-the-art tools on two data sets. As ever-growing public spectral libraries become available, spectral library search can play an important role in spectrum-centric DIA data analysis. Furthermore, integrating Calibr’s spectral library search results with sequence database search results can further improve spectrum-centric identification of DIA data.

## Supplementary Information

Supplementary Information.

## Data Availability

Calibr executable files, sample data sets, and user manual are freely available at https://ms.iis.sinica.edu.tw/COmics/Software_CalibrWizard.html and https://sourceforge.net/projects/comics-calibr. Sample data sets were acquired from published papers and can be accessed through ProteomeXchange consortium (http://www.proteomexchange.org/) with identifier PXD003179, PXD009246, and PXD003344. The MassIVE spectral library used was downloaded from https://massive.ucsd.edu/ProteoSAFe/static/massive-kb-libraries.jsp. The protein fasta file was obtained from the UniProtKB (https://www.uniprot.org/) human proteome database.
