# Supplementary material for: Calibr improves spectral library search for spectrum-centric analysis of data independent acquisition proteomics
Source: Sci Rep. 2022 Feb 7;12:2045. doi: 10.1038/s41598-022-06026-9 (PMC8821666; doi:10.1038/s41598-022-06026-9)
Supplement: Supplementary file 1 — Supplementary Information. [file 41598_2022_6026_MOESM1_ESM.docx]

Supplementary Information

**Calibr improves spectral library search for spectrum-centric analysis of data independent acquisition proteomics**

Jen-Hung Wang^1,2,3^, Wai-Kok Choong^2^, Ching-Tai Chen^4,5,*^, and Ting-Yi Sung^2,*^

1 Bioinformatics Program, Taiwan International Graduate Program, Academia Sinica, Taipei 11529, Taiwan

2 Institute of Information Science, Academia Sinica, Taipei 11529, Taiwan

3 Institute of Biomedical Informatics, National Yang Ming Chiao Tung University, Taipei 11221, Taiwan

4 Department of Bioinformatics and Medical Engineering, Asia University, Taichung 41354, Taiwan

5 Center for Precision Health Research, Asia University, Taichung 41354, Taiwan

*Correspondence: [ctchen@asia.edu.tw](mailto:ctchen@asia.edu.tw), [tsung@iis.sinica.edu.tw](mailto:tsung@iis.sinica.edu.tw)

**Table of Contents**

**Table S1.** Formula of similarity measures and various metrics used in Calibr S-2

**Table S2.** File names and URLs of the paired DIA and DDA data sets S-4

**Table S3.** Numbers of pseudo MS2 spectra at the three quality levels given by DIA-Umpire S-6

**Table S4.** Spectral library search results of the three tools S-6

**Table S5.** Jaccard indices of SSMs and peptides identified by any two spectral library search engines S-6

**Table S6.** Numbers of SSMs and peptides obtained by Calibr and validated by Percolator using different “leave-one-out” feature sets on the helaDIA data set S-7

**Table S7.** Numbers of SSMs and peptides obtained by Calibr and validated by Percolator using different “top four-plus-one” feature sets on the helaDIA data set S-8

**Table S8.** Comparison between the identification results of searching against the DDA-based library and searching against the MassIVE spectral library ……………………………………….……………………………………………S-9

**Figure S1.** Comparison of different combinations of IP and UPS on the samonDIA data set S-10

**Figure S2.** Comparison of different combinations of IP and UPS on the MCF7phosDIA data set S-10

**Figure S3.** Pairwise similarity of Percolator’s SVM scores among the three search engines on the three DIA data sets S-11

**Figure S4.** Comparison of Percolator’s SVM scores of common SSMs identified by Calibr and SpectraST with changed parameters or features in Calibr searching ………………………………………………………………………………….S-12

**Figure S5.** Distribution of Percolator’s SVM scores of search results obtained by Calibr and SpectraST on the samonDIA data set. S-13

**Figure S6.** Distribution of Percolator’s SVM scores of search results obtained by Calibr and SpectraST on the MCF7phosDIA data set S-13

**Figure S7.** Two query spectra in the helaDIA data set as examples to show the efficacy of libc_cosSim as a feature of the SSMs for Percolator validation S-14

**Figure S8.** Comparison of the PSMs obtained by using three database search engines, using one spectral library search engine, and combining the four search engines ……………………………………………………………………..…. S-16

**Figure S9.** The main interfaces of CalibrWizard for performing spectral library searching and validation………….... S-17

| **Similarity measure** | **Formula** | **Description** |
| --- | --- | --- |
| dotProduct | $\mathrm{dotProduct}\left( Q,L \right)= \frac{I_{Q}\bullet I_{L}}{\left\Vert I_{Q} \right\Vert\times\left\Vert I_{L} \right\Vert}$ | $I_{Q}$: the vector of the intensities of all the peaks (i.e., bins with positive intensities) of the query spectrum.  $I_{L}$: the vector of the intensities of all the peaks of the library spectrum. |
| Xcorr  (cross correlation) | $\mathrm{Xcorr}\left( Q, L \right)= I_{Q} ˙(I_{L}-\frac{1}{150} \sum_{\tau=-75,\tau\neq0}^{\tau=75} I_{L}\left[ \tau\right])$ | $I_{L}\left[ \tau\right]$: the vector of peak intensities of library spectrum L shifted by τ Da. |
| libc_cosSim  (library-centric cosine similarity) | $libc\_cosSim\left( Q,L \right)= \frac{{Proj(I}_{Q}, I_{L})˙I_{L}}{\left\Vert{Proj(I}_{Q}, I_{L}) \right\Vert\times\left\Vert I_{L} \right\Vert}$ | ${Proj(I}_{Q}, I_{L})=\{I_{\mathrm{Qi}}\mathrm{if}I_{\mathrm{Li}}>0, and 0 otherwise:for all peaks i\}$. |
| PCC  (Pearson correlation coefficient) | $\mathrm{PCC}\left( Q,L \right)= \frac{{\Delta I}_{Q}˙\Delta I_{L}}{\left\Vert{\Delta I}_{Q} \right\Vert\times\left\Vert\Delta I_{L} \right\Vert}$ | ${\Delta I}_{Q}$: the vector of peak intensities subtracted by the average intensity in the query spectrum.  ${\Delta I}_{L}$: the vector of peak intensities subtracted by the average intensity in the library spectrum. |
| HGT  (hypergeometric test score) | $\mathrm{HGT}\left( Q, L \right)= -log[\sum_{i>CM}^{n_{L}} \frac{\binom{n_{L}}{i}\binom{N_{Q}-n_{L}}{n_{L}-i}}{\binom{N_{Q}}{n_{L}}}]$ | $N_{Q}$: the total number of occupied and unoccupied peaks in the query spectrum  $n_{L}$: the number of peaks in the library spectrum.  CM: the number of matched peaks. |
| KT  (Kendall-Tau coefficient) | $\mathrm{KT}\left( Q, L \right)= \frac{\mathrm{CP}_{Q,L}- \mathrm{DP}_{Q,L}}{n(n-1)/2}$ | $\mathrm{CP}_{Q,L}$: the number of concordant pairs of query and library spectra.  $\mathrm{DP}_{Q,L}$: the number of discordant pairs of query and library spectra.  n: the number of matched peaks in ranking. |
| deltaD | $\mathrm{deltaD}\left( Q, L_{1}, L_{2} \right)=\frac{\mathrm{score}\left( Q,L_{1} \right)-score(Q,L_{2})}{\mathrm{score}\left( Q,L_{1} \right)}$ | score: the similarity measure used to determine SSMs, which is dot product in this study.  $L_{1}$: the best hit, i.e., the library spectrum having the highest similarity measure with the query spectrum.  $L_{2}$: the second-best hit, i.e., the library spectrum having the second highest similarity measure with the query spectrum. |
| dotBias  (dot bias) | $dotBias\left( Q,L \right)= \frac{1}{dotProduct(Q,L)}\sqrt{\frac{I_{Q}^{2}\bullet I_{L}^{2}}{\left\Vert I_{Q}^{2} \right\Vert\times\left\Vert I_{L}^{2} \right\Vert}}$ | The formula is obtained from the original paper of SpectraST by H. Lam et al. (2007). |
| penalty  (depending on dotBias) | 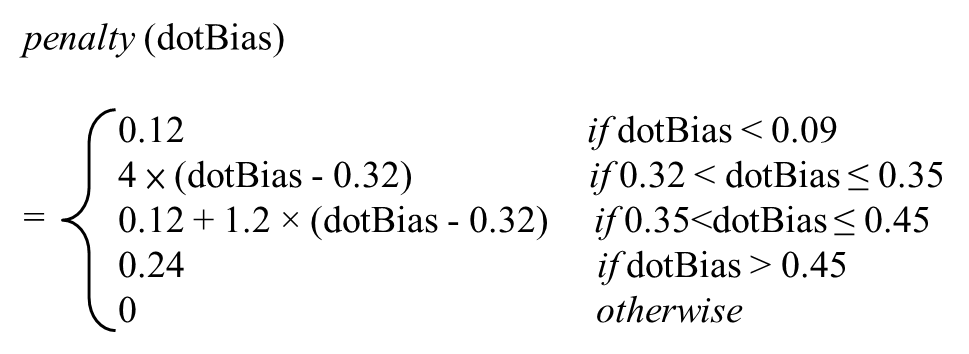 | It is defined in TPP (Trans-Proteomic Pipeline) released-5.1.0 source code available at  <https://sourceforge.net/p/sashimi/code/HEAD/tree/tags/release_5-1-0/src/Search/SpectraST/SpectraSTSimScores.cpp#l92>  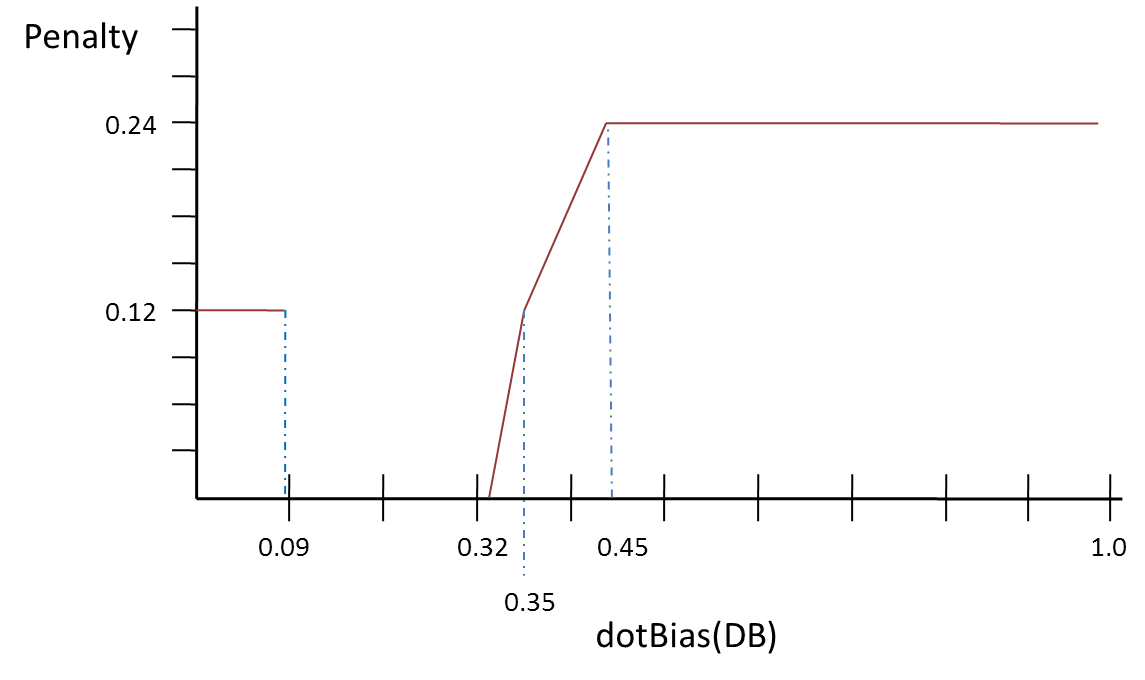 |
| Fval | $\mathrm{Fval}\left( Q, L \right)= 0.6\times dotProduct\left( Q, L \right)+ 0.4\times d\mathrm{eltaD}\left( Q, L, L_{2} \right)+ penalty$ | The formula is obtained from the original paper of SpectraST by H. Lam et al. (2007). |

**Table S1. Formula of similarity measures and various metrics used in Calibr.**

Q represents a query spectrum and L represents a library spectrum.

**Reference:** Lam, H.; Deutsch, E. W.; Eddes, J. S.; Eng, J. K.; King, N.; Stein, S. E.; Aebersold, R. Development and Validation of a Spectral Library Searching Method for Peptide Identification from MS/MS. *PROTEOMICS* **2007**, *7* (5), 655–667. <https://doi.org/10.1002/pmic.200600625>.

| **File name** | **Data set** | **URL** | **Type** | **Description** |
| --- | --- | --- | --- | --- |
| Hela1ug_QC_Middle_DDA_150226_01_150227005127.raw | helaDDA | <ftp://ftp.pride.ebi.ac.uk/pride/data/archive/2016/06/PXD003179/Hela1ug_QC_Middle_DDA_150226_01_150227005127.raw> | RAW | DDA |
| Hela1ug_QC_Middle_DDA_150226_02_150227032618.raw | helaDDA | <ftp://ftp.pride.ebi.ac.uk/pride/data/archive/2016/06/PXD003179/Hela1ug_QC_Middle_DDA_150226_02_150227032618.raw> | RAW | DDA |
| Hela1ug_QC_Middle_DDA_150226_03_150227060105.raw | helaDDA | <ftp://ftp.pride.ebi.ac.uk/pride/data/archive/2016/06/PXD003179/Hela1ug_QC_Middle_DDA_150226_03_150227060105.raw> | RAW | DDA |
| Hela1ug_QC_Middle_DIA_10Da_150226_01.raw | helaDIA | <ftp://ftp.pride.ebi.ac.uk/pride/data/archive/2016/06/PXD003179/Hela1ug_QC_Middle_DIA_10Da_150226_01.raw> | RAW | DIA |
| Hela1ug_QC_Middle_DIA_10Da_150226_02.raw | helaDIA | <ftp://ftp.pride.ebi.ac.uk/pride/data/archive/2016/06/PXD003179/Hela1ug_QC_Middle_DIA_10Da_150226_02.raw> | RAW | DIA |
| Hela1ug_QC_Middle_DIA_10Da_150226_03.raw | helaDIA | <ftp://ftp.pride.ebi.ac.uk/pride/data/archive/2016/06/PXD003179/Hela1ug_QC_Middle_DIA_10Da_150226_03.raw> | RAW | DIA |
| samon_D1801_035.raw | samonDDA | <ftp://ftp.pride.ebi.ac.uk/pride/data/archive/2019/04/PXD009246/samon_D1801_035.raw> | RAW | DDA |
| samon_D1801_038.raw | samonDDA | <ftp://ftp.pride.ebi.ac.uk/pride/data/archive/2019/04/PXD009246/samon_D1801_038.raw> | RAW | DDA |
| samon_D1801_041.raw | samonDDA | <ftp://ftp.pride.ebi.ac.uk/pride/data/archive/2019/04/PXD009246/samon_D1801_041.raw> | RAW | DDA |
| samon_D1801_034.raw | samonDIA | <ftp://ftp.pride.ebi.ac.uk/pride/data/archive/2019/04/PXD009246/samon_D1801_034.raw> | RAW | DIA |
| samon_D1801_037.raw | samonDIA | <ftp://ftp.pride.ebi.ac.uk/pride/data/archive/2019/04/PXD009246/samon_D1801_037.raw> | RAW | DIA |
| samon_D1801_040.raw | samonDIA | <ftp://ftp.pride.ebi.ac.uk/pride/data/archive/2019/04/PXD009246/samon_D1801_040.raw> | RAW | DIA |
| MCF7_phos_DDA_1.raw | MCF7phosDDA | <ftp://massive.ucsd.edu/MSV000079423/raw/MCF7_phos_DDA_1.raw> | RAW | DDA |
| MCF7_phos_DDA_2.raw | MCF7phosDDA | <ftp://massive.ucsd.edu/MSV000079423/raw/MCF7_phos_DDA_2.raw> | RAW | DDA |
| MCF7_phos_DDA_3.raw | MCF7phosDDA | <ftp://massive.ucsd.edu/MSV000079423/raw/MCF7_phos_DDA_3.raw> | RAW | DDA |
| MCF7_phos_DDA_4.raw | MCF7phosDDA | <ftp://massive.ucsd.edu/MSV000079423/raw/MCF7_phos_DDA_4.raw> | RAW | DDA |
| MCF7_phos_DIA_1.raw | MCF7phosDIA | <ftp://massive.ucsd.edu/MSV000079423/raw/MCF7_phos_DIA_1.raw> | RAW | DIA |
| MCF7_phos_DIA_2.raw | MCF7phosDIA | <ftp://massive.ucsd.edu/MSV000079423/raw/MCF7_phos_DIA_2.raw> | RAW | DIA |
| MCF7_phos_DIA_3.raw | MCF7phosDIA | <ftp://massive.ucsd.edu/MSV000079423/raw/MCF7_phos_DIA_3.raw> | RAW | DIA |
| MCF7_phos_DIA_4.raw | MCF7phosDIA | <ftp://massive.ucsd.edu/MSV000079423/raw/MCF7_phos_DIA_4.raw> | RAW | DIA |

**Table S2.** **File names and URLs of the paired DIA and DDA data sets**

The DDA data sets were used to build the sample-specific DDA-based spectral libraries for spectral library searching of the corresponding DIA data sets.

| **Data set** | **helaDIA** | **samonDIA** | **MCF7phosDIA** |
| --- | --- | --- | --- |
| Q1 | 218,732 | 204,018 | 74,362 |
| Q2 | 263,257 | 379,906 | 105,371 |
| Q3 | 71,850 | 305,444 | 88,328 |
| Total | 553,839 | 889,368 | 268,061 |

**Table S3.** **Numbers of pseudo MS2 spectra at the three quality levels given by DIA-Umpire.**

The quality levels of the spectra were used as features of SSMs for Percolator validation in this study.

| **Data set** | **helaDIA** | | **samonDIA** | | **MCF7phosDIA** | |
| --- | --- | --- | --- | --- | --- | --- |
|  | **SSMs** | **peptides** | **SSMs** | **peptides** | **SSMs** | **peptides** |
| **Calibr** | 80,235 | 22,910 | 80,069 | 21,415 | 28,152 | 5,657 |
| **SpectraST** | 64,932 | 19,341 | 60,992 | 17,105 | 16,939 | 3,943 |
| **Pepitome** | 43,236 | 9,492 | 63,220 | 12,641 | 23,939 | 4,120 |

**Table S4. Spectral library search results of the three tools.**

The three DIA data sets were searched against the respective DDA-based spectral libraries and validated by Percolator to report validated SSMs and validated peptides.

| **Data set** | **Search engines compared** | Jaccard index of SSMs | Jaccard index of peptides |
| --- | --- | --- | --- |
|  |  |  |  |
| helaDIA | Calibr vs SpectraST | 0.741 | 0.806 |
|  | Calibr vs Pepitome | 0.360 | 0.397 |
|  | SpectraST vs Pepitome | 0.371 | 0.436 |
| samonDIA | Calibr vs SpectraST | 0.693 | 0.766 |
|  | Calibr vs Pepitome | 0.468 | 0.556 |
|  | SpectraST vs Pepitome | 0.466 | 0.597 |
| MCF7phosDIA | Calibr vs SpectraST | 0.502 | 0.624 |
|  | Calibr vs Pepitome | 0.510 | 0.636 |
|  | SpectraST vs Pepitome | 0.414 | 0.576 |

**Table S5.** **Jaccard indices of SSMs and peptides identified by any two spectral library search engines.**

The DDA-based spectral libraries for the corresponding DIA data sets were used for spectral library searching.

| **Excluded feature** | **SSMs** | **Peptides** | **cont_SSM** | **cont_Pep** |
| --- | --- | --- | --- | --- |
| Xcorr | 78,539 | 22,352 | 0.02114 | 0.02436 |
| libc_cosSim | 79,256 | 22,584 | 0.01220 | 0.01423 |
| dotBias | 79,722 | 22,667 | 0.00639 | 0.01061 |
| QualityLevel | 79,875 | 22,773 | 0.00449 | 0.00598 |
| charge | 80,070 | 22,899 | 0.00206 | 0.00048 |
| hits_mean | 80,142 | 22,882 | 0.00116 | 0.00122 |
| precursor_mz_diff | 80,161 | 22,880 | 0.00092 | 0.00131 |
| KT | 80,162 | 22,847 | 0.00091 | 0.00275 |
| Pvalue | 80,181 | 22,894 | 0.00067 | 0.00070 |
| secondScore | 80,183 | 22,885 | 0.00065 | 0.00109 |
| hits_stdev | 80,205 | 22,818 | 0.00037 | 0.00402 |
| HGT | 80,222 | 22,943 | 0.00016 | -0.00144 |
| deltaD | 80,230 | 22,884 | 0.00006 | 0.00113 |
| penalty | 80,239 | 22,883 | -0.00005 | 0.00118 |
| PCC | 80,239 | 22,918 | -0.00005 | -0.00035 |
| dotProduct | 80,268 | 22,891 | -0.00041 | 0.00083 |
| hits_num | 80,280 | 22,907 | -0.00056 | 0.00013 |
| Fval | 80,289 | 22,911 | -0.00067 | -0.00004 |
| massdiff | 80,317 | 22,901 | -0.00102 | 0.00039 |

**Table S6.** **Numbers of SSMs and peptides obtained by Calibr and validated by Percolator using different “leave-one-out” feature sets on the helaDIA data set.**

Using all the features for Percolator validation obtained 80235 validated SSMs and 22910 validated peptides. The contribution of each feature was calculated based on the differences in the numbers of SSMs and peptides with respect to using all features; positive contribution means excluding the feature reduces the SSMs or the peptides.

| **Feature set** | **SSMs**  **(number)** | **Peptides**  **(number)** | **SSMs (percentage)** | **Peptides (percentage)** |
| --- | --- | --- | --- | --- |
| All (19) | 80,235 | 22,910 | 100.00% | 100.00% |
| Top four_only (4) | 72,948 | 21,230 | 90.92% | 92.67% |
| Top four + secondScore (5) | 76,378 | 22,105 | 95.19% | 96.49% |
| Top four + dotProduct (5) | 75,139 | 21,658 | 93.65% | 94.54% |
| Top four + deltaD (5) | 75,043 | 21,660 | 93.53% | 94.54% |
| Top four + hits_mean (5) | 74,715 | 21,620 | 93.12% | 94.37% |
| Top four + hits_stdev (5) | 74,445 | 21,378 | 92.78% | 93.31% |
| Top four + Fval (5) | 73,256 | 21,320 | 91.30% | 93.06% |
| Top four + Kendall-Tau (5) | 73,245 | 21,273 | 91.29% | 92.85% |
| Top four + charge (5) | 73,120 | 21,202 | 91.13% | 92.54% |
| Top four + precursor_mz_diff (5) | 73,045 | 21,211 | 91.04% | 92.58% |
| Top four + massdiff (5) | 73,028 | 21,190 | 91.02% | 92.49% |
| Top four + HGT (5) | 72,989 | 21,255 | 90.97% | 92.78% |
| Top four + penalty (5) | 72,939 | 21,181 | 90.91% | 92.45% |
| Top four + hits_num (5) | 72,939 | 21,149 | 90.91% | 92.31% |
| Top four + PCC (5) | 72,897 | 21,178 | 90.85% | 92.44% |
| Top four + Pvalue (5) | 72,633 | 21,136 | 90.53% | 92.26% |

**Table S7.** **Numbers of SSMs and peptides obtained by Calibr and validated by Percolator using different “top four-plus-one” feature sets on the helaDIA data set.**

Top four features are Xcorr, libc_cosSim, dotBias, and QualityLevel. Percentage of SSMs and peptides were determined by the identification number of using the specific “top four-plus-one” feature set for validation divided by that of using all 19 features for validation.

|  | | **SSMs** | | | | **Peptides** | | | | |
| --- | --- | --- | --- | --- | --- | --- | --- | --- | --- | --- |
| **Data set** | **Tool** | **DSL** | **MSL** | **Increase** | **Increase (%)** | **DSL** | **MSL** | **Increase** | **Increase (%)** |  |
| helaDIA | Calibr | 80235 | 94488 | 14253 | 17.76% | 22910 | 30975 | 8065 | 35.20% |  |
|  | SpectraST | 64932 | 71859 | 6927 | 10.66% | 19341 | 24733 | 5392 | 27.87% |  |
|  | Pepitome | 43236 | 65033 | 21797 | 50.41% | 9492 | 18276 | 8784 | 92.54% |  |
| samonDIA | Calibr | 80069 | 71779 | -8290 | -10.35% | 21415 | 23338 | 1923 | 8.97% |  |
|  | SpectraST | 60992 | 51538 | -9454 | -15.50% | 17105 | 17840 | 735 | 4.29% |  |
|  | Pepitome | 63220 | 47895 | -15325 | -24.24% | 12641 | 11770 | -871 | -6.89% |  |

**Table S8. Comparison between the identification results of searching against the DDA-based library and searching against the MassIVE spectral library.**

DSL denotes searching against DDA-based spectral library; MSL denotes searching against MassIVE spectral library. Increase is calculated by SSM or peptide number of MSL minus that of DSL. Increase (%) is calculated by the corresponding increase divided by SSM or peptide number of DSL.

**
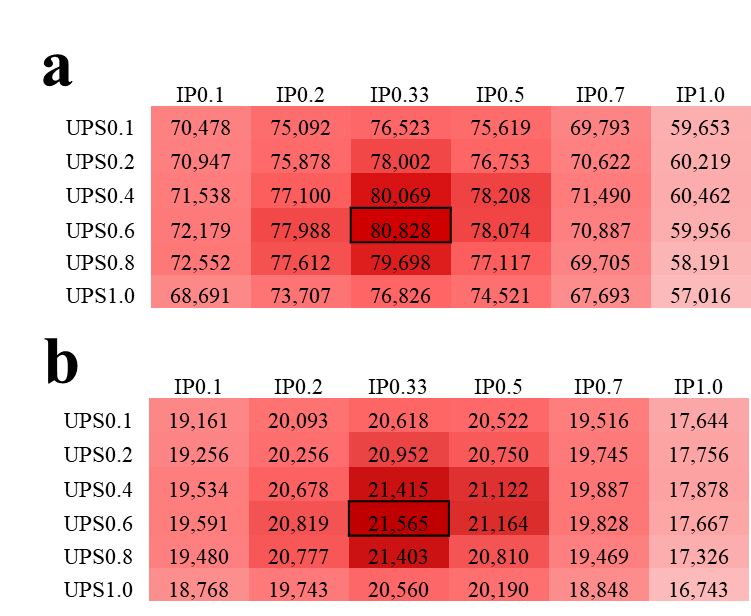
**

**Figure S1. Comparison of different combinations of intensity power (IP) and unassigned peak scaling (UPS) in terms of identification results of searching the samonDIA data set using Calibr.**

(**a**) The number of validated SSMs. (**b**) The number of validated peptides. Validation was performed using Percolator. The largest value is marked by a black box.


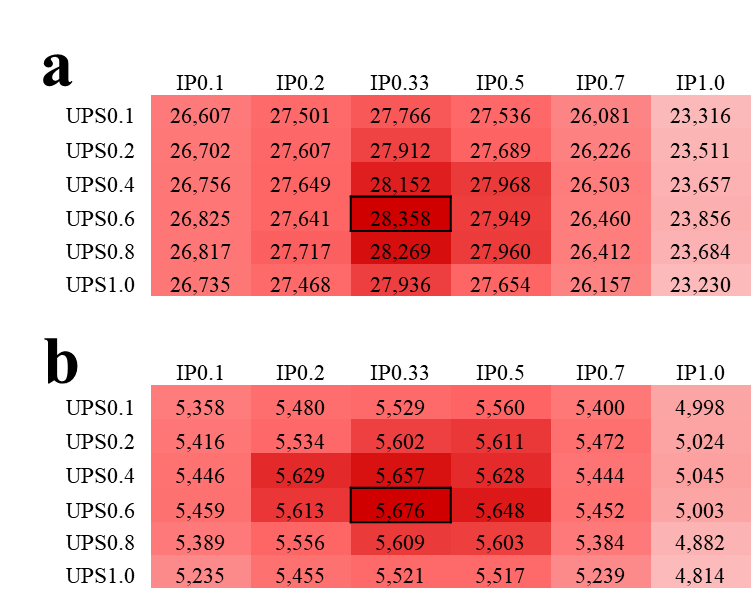


**Figure S2. Comparison of different combinations of intensity power (IP) and unassigned peak scaling (UPS) in terms of identification results of searching the MCF7phosDIA data set using Calibr.**

(**a**) The number of validated SSMs. (**b**) The number of validated peptides. Validation was performed using Percolator. The largest value is marked by a black box.


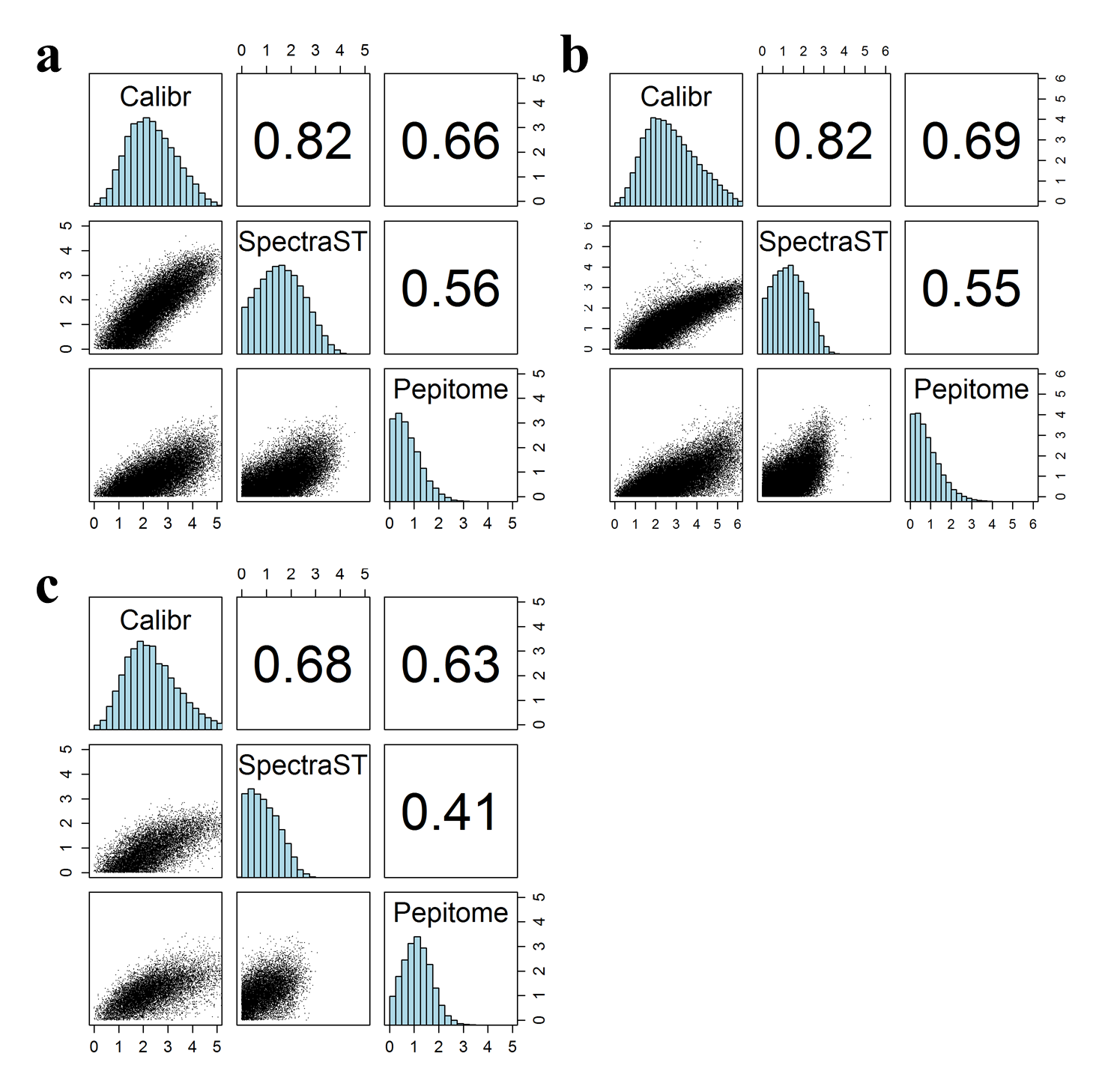


**Figure S3.** **Pairwise similarity of Percolator’s SVM scores among the three search engines on the three DIA data sets.**

Only the shared SSMs among the three search engines are used for calculating correlations. (**a**) helaDIA data set. (**b**) samonDIA data set. (**c**) MCF7phosDIA data set.


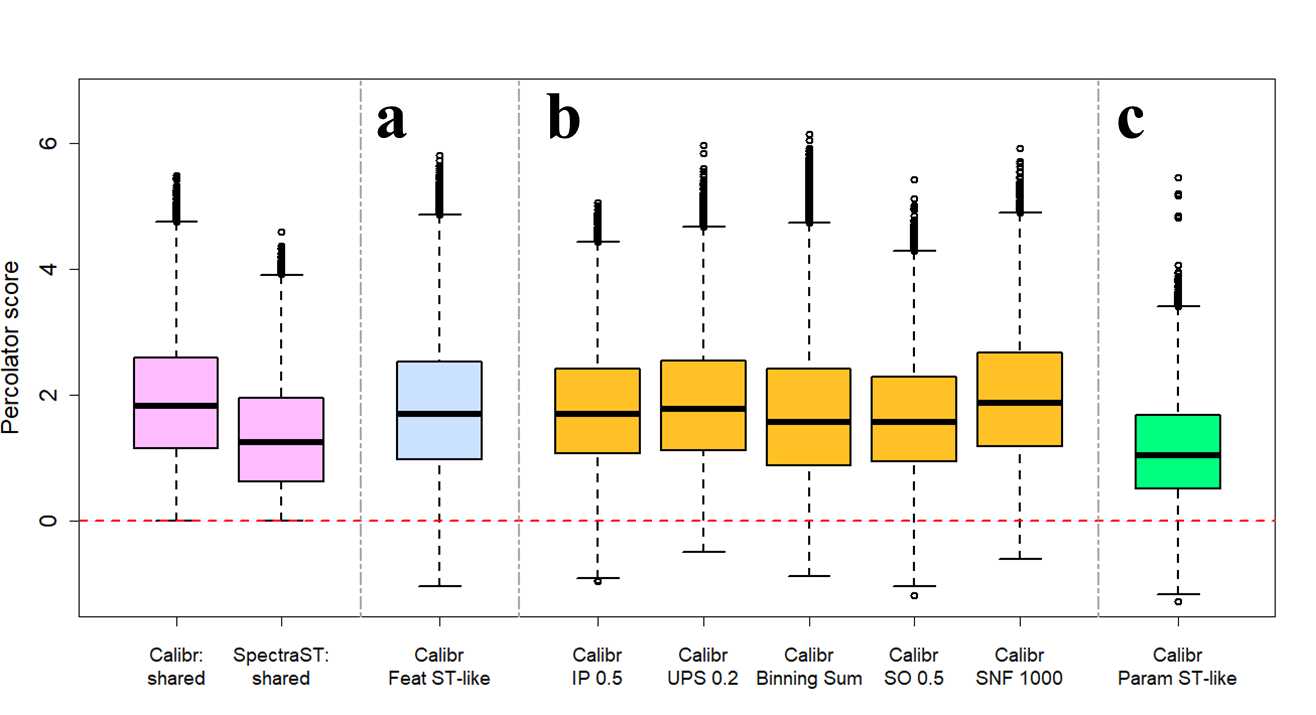


**Figure S4. Comparison of Percolator’s SVM scores of common SSMs identified by Calibr and SpectraST with changed parameters or features in Calibr searching.**

The two pink boxplots at left side are the SVM scores of commonly identified SSMs by Calibr and SpectraST, same as in Figure 5. The other boxplots are the updated scores of the common SSMs in Calibr’s results when its parameters for preprocessing spectra or features generated for validation are respectively changed. (**a**) The scores of the common SSMs of Calibr when the exclusive features, Xcorr, libc_cosSim, HGT, Kendall-Tau coefficient, Pearson correlation coefficient, penalty, and second best dot product are omitted from validation. (**b**) The scores of common SSMs of Calibr with replacing each individual optimized parameter for spectra preprocessing with SpectraST default value and validated with all the Calibr-generated features for validation. Note that the SSM sets are not completely the same as original sets (the pink boxes) since the spectra with changed individual preprocessing parameters may match to different spectra in the spectral library. (**c**) The scores of common SSMs of Calibr using all the preprocessing parameters same as SpectraST’s default parameters and validated with all features.

Feat ST-like: Calibr using the features in common with SpectraST for Percolator validation, i.e., excluding the exclusive features generated by Calibr.

IP 0.5: intensity power of 0.5.

UPS 0.2: unassigned-peak scaling of 0.2.

Binning Sum: binning using Sum instead of using Max.

SO 0.5: spillover with fraction 0.5.

SNF 1000: SNfilter with factor 1000.

Param ST-like: applying all the above five parameters same as the default settings of SpectraST.
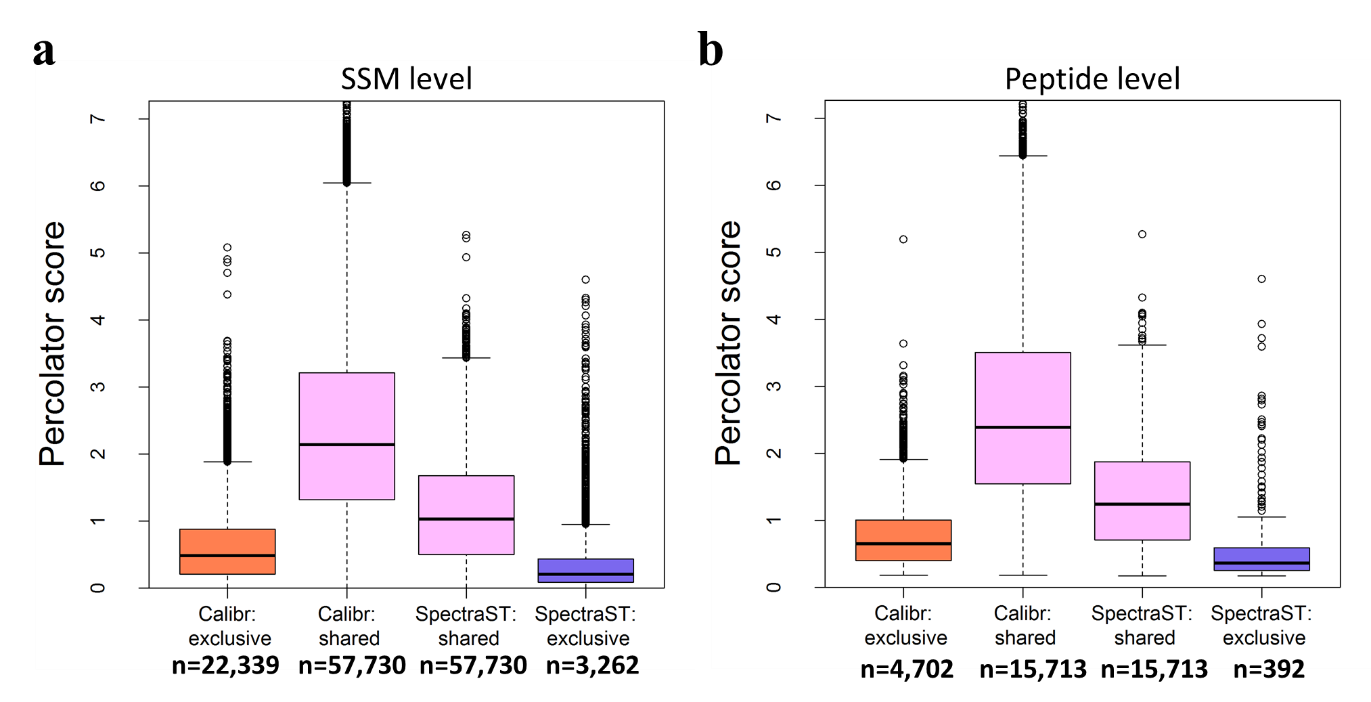


**Figure S5.** **Distribution of Percolator’s SVM scores of search results obtained by Calibr and SpectraST on the samonDIA data set.**

(**a**) SVM scores of validated SSMs. (**b**) SVM scores of validated peptides. The SSMs and peptides are grouped into commonly obtained by SpectraST and Calibr (pink boxes) and exclusively obtained by one search engine (orange box for Calibr and purple box for SpectraST).


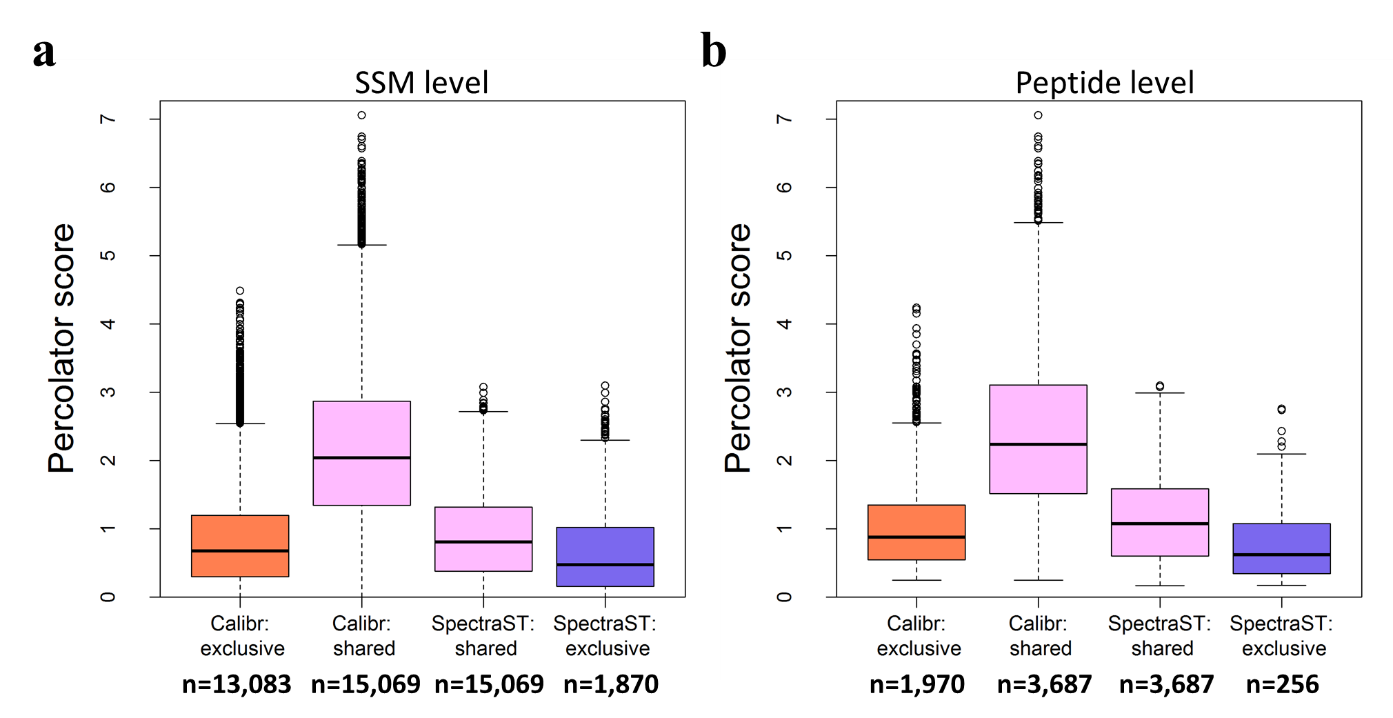


**Figure S6.** **Distribution of Percolator’s SVM scores of search results obtained by Calibr and SpectraST on the MCF7phosDIA data set.**

(**a**) SVM scores of validated SSMs. (**b**) SVM scores of validated peptides. The SSMs and peptides are grouped into commonly obtained by SpectraST and Calibr (pink boxes) and exclusively obtained by one search engine (orange box for Calibr and purple box for SpectraST).


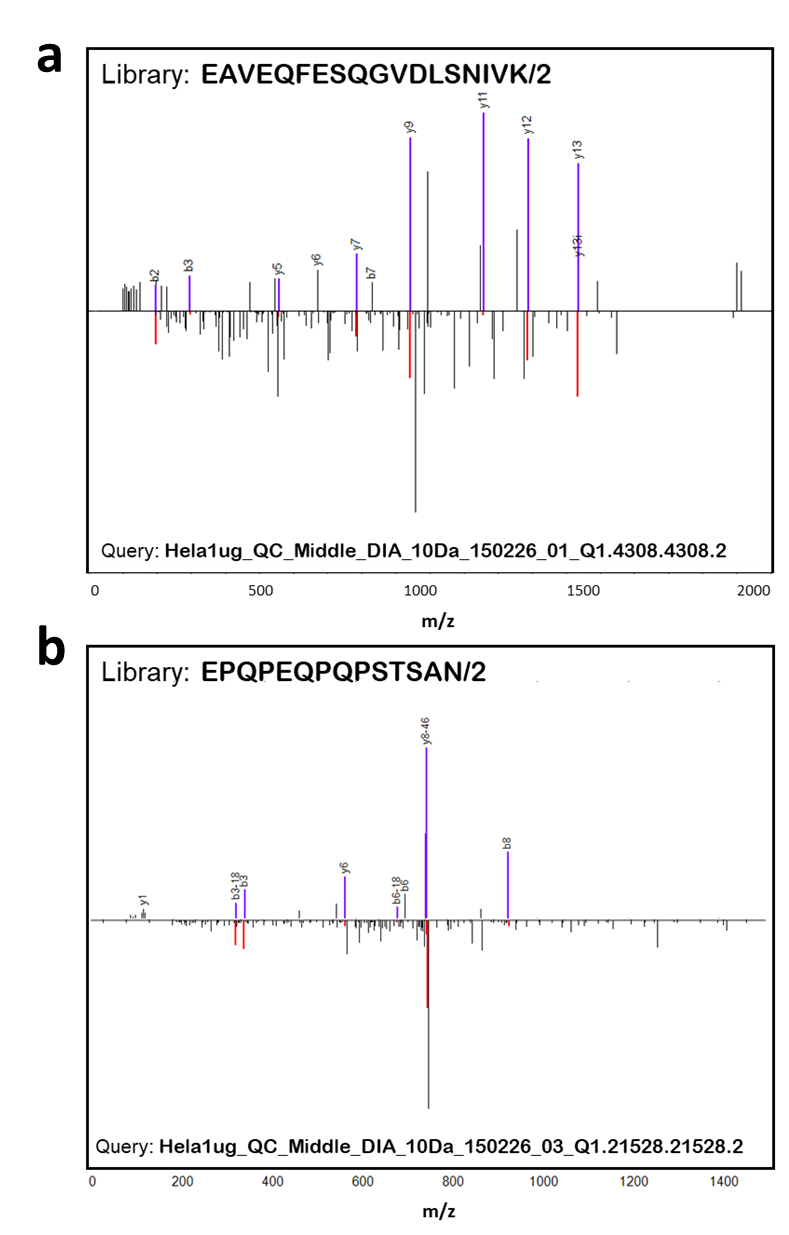


**Figure S7.** **Two query spectra in the helaDIA data set as examples to show the efficacy of libc_cosSim as a feature of the SSMs for Percolator validation.**

In each sub-figure, the query spectrum is shown at the bottom, and the matched library spectrum shown at the top. Both SSMs are identified by Calibr and not by SpectraST. To be specific, the Percolator scores of both query spectra using Calibr (1.09960 for (**a**) and 0.06220 for (**b**)) are significantly higher than the score threshold of -0.00065 corresponding to 1% FDR. The Percolator scores of both spectra using SpectraST (-0.16025 for (**a**) and -0.47021 for (**b**)) are significantly smaller than the score threshold of -0.00036 corresponding to 1% FDR. The seven similarity measures including libc_cosSim, dot product, Fval, Xcorr, PCC, HGT, and Kendall-Tau coefficient are normalized by Z-score transformation for further analysis. For subfigure (**a**), the Z-scores for the seven similarity measures are 1.597, -1.540, -1.663, -0.824, -0.924, -0.979, and -1.857, respectively. For subfigure (**b**), the Z-sores for the seven similarity measures are 1.742, -1.367, -3.586, -0.834, -0.078, -1.058, and -1.910, respectively. The large differences in Percolator scores between Calibr and SpectraST are very likely caused by the use of libc_cosSim, since it is the only similarity measure with large Z-score and is exclusively used by Calibr.


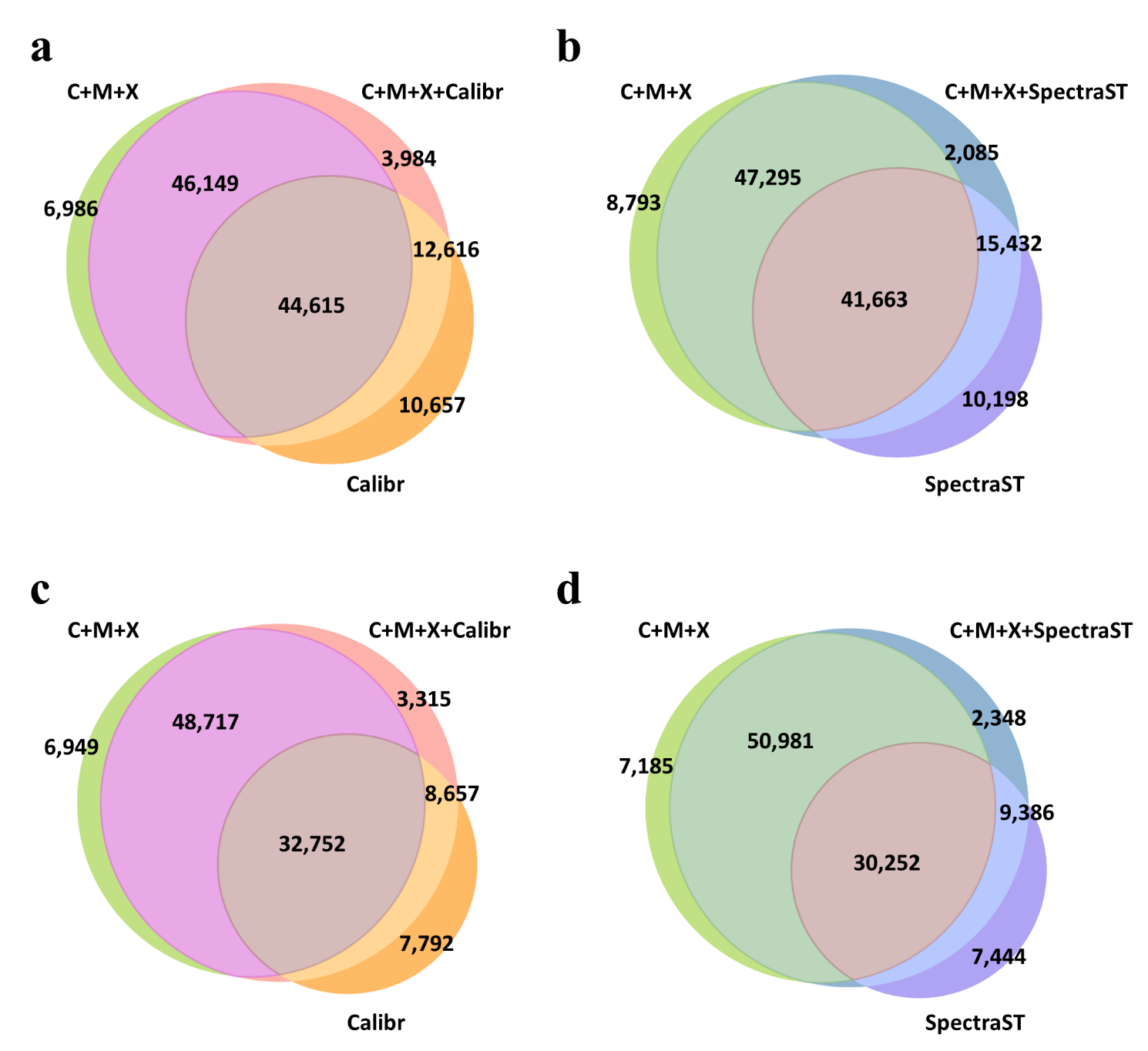


**Figure S8. Comparison of the PSMs obtained by using three database search engines, using one spectral library search engine, and combining the four search engines.**

(**a**) Venn diagram of PSMs obtained by three database search engines and Calibr from the helaDIA data set. (**b**) Venn diagram of PSMs obtained by three database search engines and SpectraST from the helaDIA data set. (**c**) Venn diagram of PSMs obtained by three database search engines and Calibr from the samonDIA data set. (**d**) Venn diagram of PSMs obtained by three database search engines and SpectraST from the samonDIA data set.

The search results of a single search engine were first validated using PeptideProphet. The multiple PeptideProphet results were further combined and refined using iProphet. The combination of the three database search engines─Comet, MS-GF+, and X!Tandem─is denoted as “C+M+X.”


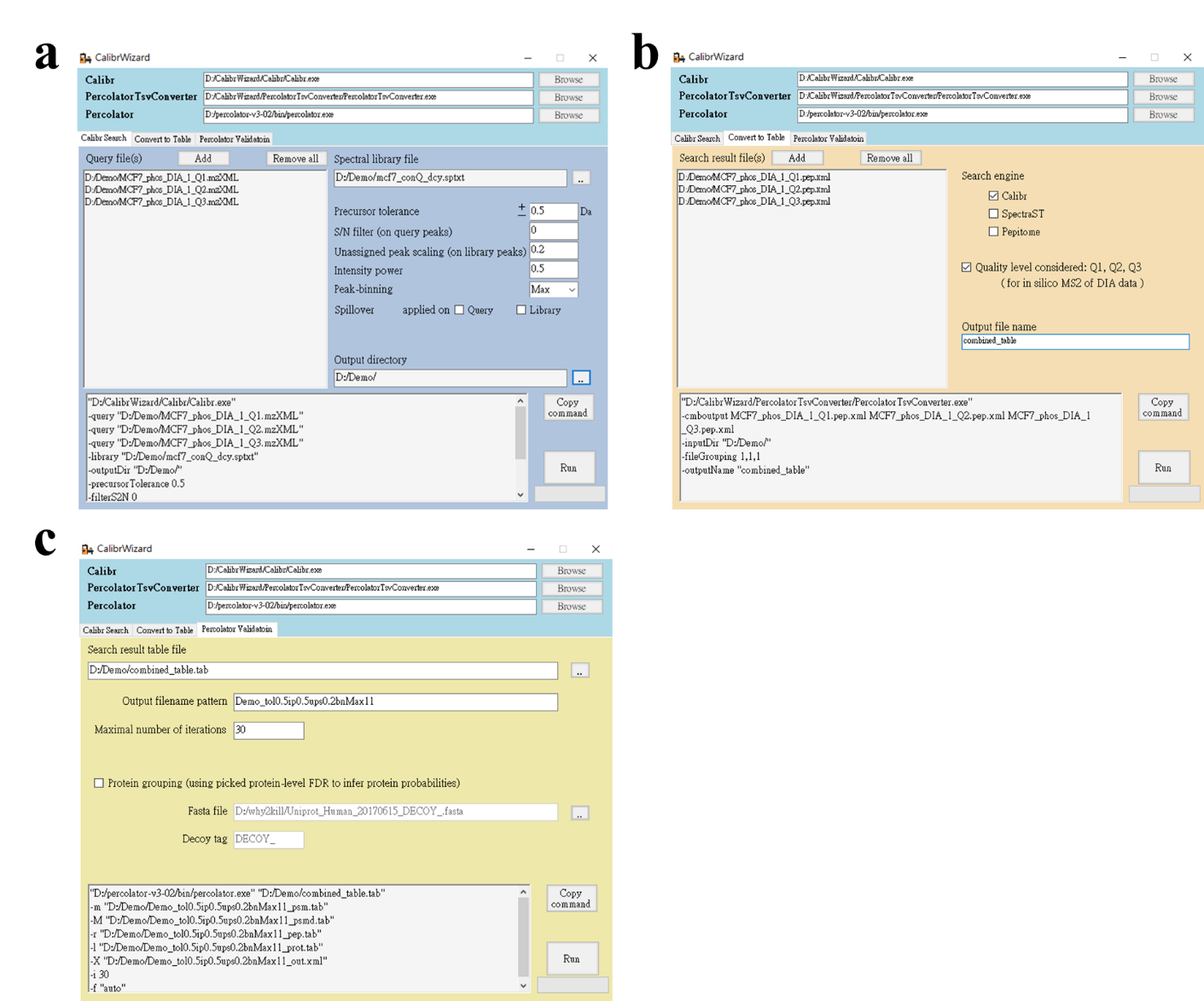


**Figure S9. The main interfaces of CalibrWizard for performing spectral library searching and validation.**

There are three tab pages in the CalibrWizard applications. (**a**) Calibr searching. (**b**) Using PercolatorTsvConverter to convert the search results to Percolator’s input format. (**c**) Percolator validation.
